# Artificial Solid Electrolyte Interphase Developed In Vitro by Tailoring Molecular Layer Deposition of a Li-Ion-Containing Electrolyte on Carbonaceous Anode Materials

**DOI:** 10.1021/acsami.5c08880

**Published:** 2025-09-16

**Authors:** Roman G. Fedorov, Jonas Schlaier, Nickolay Solomatin, Mahmud Auinat, Igor Baskin, Christian Heubner, Alexander Michaelis, Yair Ein-Eli

**Affiliations:** † Department of Materials Science and Engineering, 26747 Technion-Israel Institute of Technology, Haifa 320003, Israel; ‡ 28434 Fraunhofer Institute for Ceramic Technologies and Systems IKTS, Dresden 01277, Germany; § Institute of Materials Science, TU Dresden, Dresden 01062, Germany; ∥ Grand Technion Energy Program, Technion-Israel Institute of Technology, Haifa 320003, Israel; ⊥ Israel National Institute for Energy Storage (INIES), Technion-Israel Institute of Technology, Haifa 320003, Israel

**Keywords:** lithium-ion batteries, artificial solid–electrolyte interphase, carbonaceous anode materials, in vitro, atomic/molecular layer deposition, Li-ion electrolyte

## Abstract

Various carbonaceous anode materials have been developed to improve both the rate and capacity characteristics of Li-ion batteries (LIBs), and yet the performances of the anodes depend on the quality of the inevitable and uncontrollable growth of the solid–electrolyte interphase (SEI), resulting from the electrolyte reduction and decomposition during the initial cycles. Here, we propose the fabrication of an artificial SEI (Art-SEI), enabling tuning of specific properties, such as the chemical composition, electrochemical impedance, and thickness of the interfacial film. In this work, a genuine Art-SEI was conformally fabricated via molecular layer deposition (MLD), utilizing as one of the precursors a commercial battery electrolyte itself, with a cross-linker-functionalized film grown on the surface of the carbonaceous anode materials, along with a Li-ion source. This type of air- and moisture-stable Art-SEI possesses enhanced protective characteristics, and it mitigates the irreversible capacity loss associated with the SEI buildup during the formation cycles while substantially improving Li-ion battery cycling performances.

## Graphical abstract

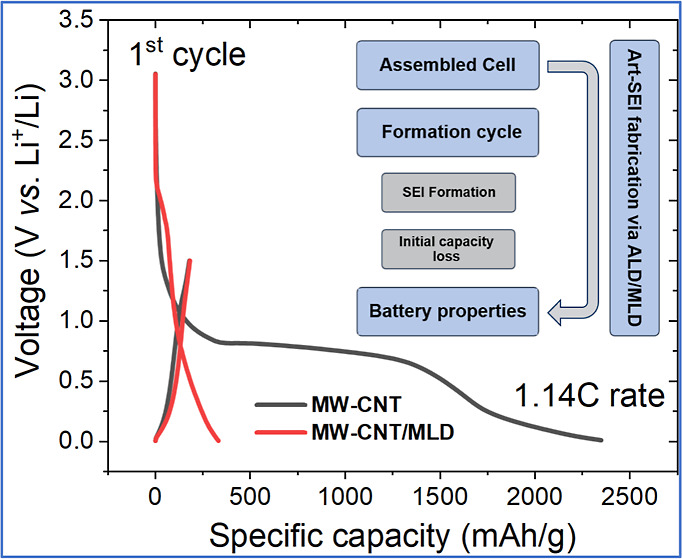

## 1 Introduction

One of the significant challenges in the field of batteries, including lithium batteries (LIBs), is the uncontrollable growth and the possibility of producing poor quality of naturally formed solid–electrolyte interphases (SEIs), which result from the reduction and decomposition of the electrolyte during initial charging–discharging cycles. The concept SEI formed on alkali metals in nonaqueous electrolytes was coined and introduced by Peled in 1979,^1^ and since then, this model has been developed by him and other groups into a standard one, describing the anode (negative electrode) interfacial phenomena also in LIBs.^2,3^ In this regard, the advantages, as well as the different challenges and the undesired processes, associated with the SEI formation and its characteristics within different anode types have been rigorously studied.^3^ One of the main strategies addressing the challenge of uncontrolled formation of the SEI is an ex situ (before LIB assembly) modification of the anode material surfaces with an artificial and tunable protective film, which provides a predictable level of durability and capacity retention over hundreds of cycles. A widely accepted term for such protective film formation is the ***artificial SEI*** (Art-SEI).^4^ Many synthetic methods have been adapted to form a robust Art-SEI on various anode materials.^5^ Accordingly, specific requirements have been formulated for each class of these materials.^6^ Moreover, the multiparameter assessment of various Art-SEIs manufacturing approaches in terms of their real-world applicability to different electrode types was also proposed.^7^

Of particular interest is the fabrication of an Art-SEI on graphite, the most used anode material in commercial LIBs, owing to its abundance in nature, exceptionally low cost, and stable and safe performance, yet holding a moderate theoretical capacity (372 mAh/g). However, the operation of modern commercial carbonaceous (Li_*x*_C_6_) anodes is far from ideal and could be greatly improved by applying an Art-SEI, which provides better protection and faster Li-ion transport as compared with naturally electrolytically formed SEI.^8^ In this regard, a clear understanding of the failure mechanism of graphite anodes in LIBs is of particular importance.^9^

Once the electrochemical potential at the graphite/electrolyte interface is gradually decreased close to the Li/Li^+^ RedOx potential (0 V vs Li/Li^+^), the Li-ion intercalation process into the layered graphite structure commences. Although even earlier than this occurs, the carbonaceous anode experiences at its surface electrolyte components electroreduction and decomposition.^10^ This is shown in Supporting Information Figure S1, presenting a slow-scan cyclic voltammetry and a constant current polarization in half-cells (vs Li metal) of two different graphite-based composite electrodes. The insoluble products of the electroreduction process adsorb at the graphite surface, forming the SEI film that defines the kinetics of subsequent Li-ion intercalation/deintercalation processes. Obviously, a lack of a smart electrolyte design or a mastered control over the constituents of the composite electrodes and electrolytes drastically impacts the SEI layer growth. Poor design and inferior buildup of the SEI inevitably would lead to degraded performance of the LIB’s anode. Such inadequate SEI formation possibly may even cause a cointercalation of solvated Li-ions, as well as considerable losses of essential active Li-ions, buried into the SEI film.^11,12^ While the former promotes the growth of interfacial resistance, the latter leads to a fading of the overall cell capacity. Moreover, if the SEI film is not conformal and/or insufficiently electrically insulating, the dissolution of solvated electrons (***e***_**sol**_^–^) occurs at potentials lower than 0.5 V (vs Li/Li^+^).^13^ Such ***e***_**sol**_^–^ can interact with organic anions in the electrolyte, solvent molecules, and other components and additives in the electrolyte, leading to its gradual degradation. Therefore, of emerging importance is the groundwork modification of the anode’s surface with the introduction of a multifunctional Art-SEI layer, since it would provide fast, reversible, and selective Li-ion intercalation/deintercalation processes through the anode interface and at the same time, it would serve as a robust barrier, preventing electron transport at its interface with the electrolyte and, importantly, it will minimize the overall “cost” in Li-ion consumption from the cathode material. Implementation of a simple, adaptable, and scalable Art-SEI will allow an actual increase in the LIB’s overall reversible capacity and consequently the cell energy density.

Designing an Art-SEI on the graphite electrode implies addressing the following requirements: (i) (electro)­chemical stability against electrolyte components to prevent the electroreduction of the latter; (ii) structural durability and elasticity to withstand volume changes during cycling, as well as preventing graphite exfoliation; (iii) selective Li-ion conductivity to suppress solvate shell cointercalation; and (iv) fast ionic transport parallel to basal planes of graphite, enabling high-power performance.^5,7^ To achieve these prerequisites, various synthetic routes for the development of an Art-SEI on graphite have been suggested. Among them, the most effective are (electro)­chemical grafting,^14^ electrodeposition,^15^ as well as atomic/molecular layer deposition (ALD/MLD).^16^ While grafting and electrodeposition strategies provide simple, fast, and cost-effective coating of the bulk graphite anodes, ALD/MLD methods enable the formation of a uniform coating of powdered graphite materials with an atomic/molecular resolution.^7^

Indeed, the application of ALD and MLD methods for designing an Art-SEI on graphite and other carbonaceous materials is of emerging interest, enabling scalable coating protocols at both active powder^17^ and composite electrode^18^ levels. In this regard, it should be emphasized that efficient Art-SEI engineering and its construction with inorganic, organic, and hybrid (metal–organic) coatings is not limited only to flat substrates (electrodes) but also applicable to individual particles, with high conformity, uniformity, and self-limiting growth, thereby providing a precise control over the film thickness and composition. This is the main distinctive advantage of the ALD/MLD approach, as shown in Scheme 1. Specifically, various carbonaceous microparticles and nanomaterials, used as active anode materials in LIBs, were uniformly modified with artificial thin layers with the use of thermal ALD/MLD,^19^ equipped with a dynamic flow system, namely, fluidized bed reactor (FBR).^20^ Among the subjected materials are multiwalled carbon nanotubes (MW-CNTs),^21^ meso-carbon microbeads (MCMB),^22^ and graphene nanosheets (GNS).^23^

**Scheme 1:**
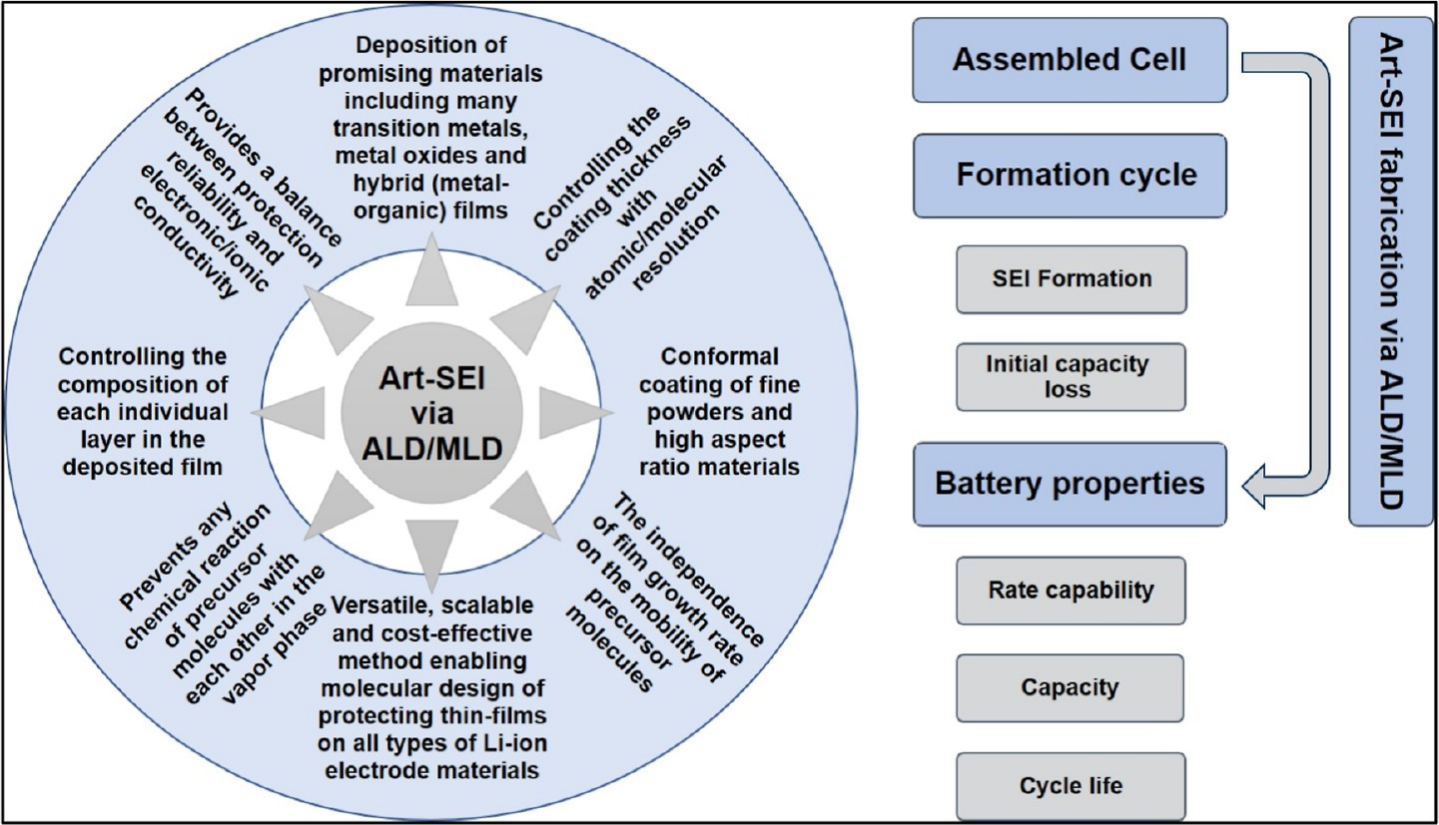
A Scheme Describing the Idea of Building Multiproperty Art-SEI by ALD/MLD

Importantly, all previous efforts to form an Art-SEI on graphite powder and carbonaceous nanoparticles via ALD/MLD have been focused exclusively on inorganic materials, i.e., metal oxides and metal salts (fluorides, phosphates, etc.), and were overlooked by organic and hybrid (metal–organic) compounds that have shown superior performances when deposited via other methods including chemical, electrochemical, and polymer grafting, as well as wet chemistry.^7^ Indeed, due to increased elasticity and flexibility, various organic and hybrid thin films are able to accommodate the anisotropic volume expansion and strains arising at sharp edges and corners of small carbonaceous particles. This becomes especially important in light of the fact that in the past decade, different flexible organometallic MLD coatings have already been applied quite successfully for another type of anode material, namely, Si.^24,25^

Here, we present the fabrication of a robust and water- and air-stable Art-SEI film made mainly of aluminum-cross-linked alkyl carbonates on the surface of individual particles of the MCMB powder and MW-CNT tissues using the thermal MLD method. Both the (electro)­chemical stability and robustness of the formed Art-SEI against the electrolyte components and the interaction with the open environment (air and humidity) and water-based binder processing are achieved by the application of a commercial electrolyte and a cross-linker as MLD precursors. While the former eventually assures a complete compatibility and adaptability of the Art-SEI toward the electrolyte at a molecular level, the latter firmly binds the constituents of a growing MLD film, forming a single structure of high strength, thereby preventing the possibility of subsequent oxidation/hydration. Moreover, the Art-SEI was additionally (pre)­lithiated during the MLD process by applying the Li-ion precursor, enabling a significant mitigation of the extremely high irreversible capacity loss in the formation cycles of the MW-CNTs/Art-SEI anodes, as well as higher initial charge capacity in the case of Art-SEI/MCMB anodes.

## 2 Experimental Section

### 2.1 Synthesis of Art-SEI Using MLD

The fabrication of MLD coating on a 5 μm MW-CNT tissue (areal density 2 mg/cm^2^, Tortech Nano Fibers, Israel and UK) and on MCMB graphite powder (17.6 μm average particle size, MTI Corporation, USA) was performed at Technion using commercial ALD (TFS-200-189, Beneq, Finland) in fixed bed and fluidized bed reactors, correspondingly. The fluidized bed reactor was equipped with a vibration generator, providing extensive powder mixing in all directions and therefore promoting more uniform and conformal coating. The carrier gas was N_2_ (99.997%), and the gas flow rates were 60 and 100 sccm when purging. The reactor conditions were as follows: 15 mbar pressure and 225 °C temperature. Trimethylaluminum (TMA, Strem Chemicals Inc.) was preloaded in a 50 mL Swagelok container at a temperature of 25 °C. A 1.0 M solution of lithium hexafluorophosphate (LiPF_6_) in ethylene carbonate and dimethyl carbonate, EC/DMC = 50/50 (v/v) (LP-30, battery grade, Merck) was also preloaded as a complex MLD-precursor at 25 °C. Lithium *tert*-butoxide (LiO^*t*^Bu, Sigma-Aldrich) was preloaded at a hot source temperature of 160 °C.

A batch of 30 g of the MCMB material powder was placed in the fluidized bed reactor for coating. Each MLD cycle (Table 1) consisted of the following steps: TMA pulse for 0.5 s, then purging for 10 s, followed by LiO^*t*^Bu pulse for 10 s, then again purging for 30 s, followed by LP-30 pulse for 1 s and purging for 30 s. Such an MLD protocol was sequentially cycled 20–80 times with the aim of pinpointing the optimal MLD cycles.

**Table 1:** MLD Cycle Protocol Applied to Modify the Active Anode Materials (MW-CNTs and MCMB) with the Art-SEI

cross-linker	purging	Li precursor	purging	Art-SEI precursor	purging
TMA—0.5 s	10 s	LiO^*t*^Bu—10 s	30 s	LP-30—1 s	30 s

Also, the Al_2_O_3_-ALD-coated MCMB graphite powder was used as one of the reference materials. In this case, the modification of the MCMB graphite material with the Al_2_O_3_-ALD coating was performed using the optimized process, which was well documented by us earlier.^26,27^ Specifically, a total of 40 ALD-cycles were performed, yielding an Al_2_O_3_ layer of approximately 3.2 nm thick based on a calibrated growth rate of ∼0.8 Å per cycle.

The feasibility of hypothetical chemical transformations that could potentially occur during MLD deposition of the Art-SEI was quantitatively assessed by calculating the changes in the internal energy (Δ*E*) of certain molecular systems during chemical transformations. Specifically, quantum chemical modeling of all molecules was carried out using the ORCA software package^28^ in two stages. In the first stage, the initial geometry of each molecule was optimized using the semiempirical quantum chemical method XTB (extended tight-binding) based on the GFN2-xTB model.^29^ In the second stage, the energy of the molecule at the XTB-optimized fixed geometry was calculated using the electron density functional theory (DFT) utilizing the Becke–Perdew (BP) exchange–correlation functional^30,31^ with the resolution of the identity (RI) approximation with the def2-SVP basis set^32^ used for all atoms and the def2/J auxiliary basis for the RI approximation.^33^

### 2.2 Material Characterization

High-resolution scanning electron microscopy (Zeiss Ultra-Plus FEG-SEM, Germany) was utilized for the surface morphology analyses of pristine and MLD-coated MW-CNT tissues and MCMB graphite powder samples. The inner layer morphology of the MLD-coated MW-CNT sample was obtained after removing the upper layer of the MW-CNT tissue by tweezers, lifting from the edge. The removed upper layer was approximately 2 μm thick. In addition, prior to the SEM measurements, monolayer arrays of the pristine and MLD-coated MCMB graphite particles have been supported on an adhesive copper tape having high electrical conductivity to get consistent results and avoid possible confusion and artifacts that could occur if a carbon tape support was used. The TEM structural and elemental cross-sectional analysis of the MLD film and the SEI layer on the MCMB graphite powder and on graphite species extracted from MCMB/MLD and MCMB/Al_2_O_3_ electrodes that had been subjected to 100 charge–discharge cycles at 0.1 C rate (postmortem analysis) was accomplished using a transmission electron microscope (Titan Themis G^2^ 60-300, FEI/Thermo Fisher, USA) equipped with a scanning transmission electron microscope (STEM) system including a high-angle annular dark-field (HAADF) and energy-dispersive X-ray spectroscopy (EDS) system. Prior to the cross-sectional analysis of the variously modified MCMB graphite species, their surface was initially coated with a 90% Pt–10% C protective mask using the ion-deposition technique. In some cases, the MCMB graphite surface was initially coated with the 80% Au–20% Pd anticharging thin coating using the sputtering method. Next, cross-sectional ion beam milling was applied using the plasma-focused ion beam (PFIB) workstation (Helios 5, Thermo Fisher Scientific, USA) to obtain the cross-sectional interface (TEM lamella), which was further analyzed with TEM. X-ray photoelectron spectroscopy (XPS) measurements were performed using a Kratos AXIS Supra spectrometer (Kratos Analytical Ltd., Manchester, U.K.) with an Al Kα monochromatic radiation X-ray source (1486.6 eV). The XPS spectra were acquired with a takeoff angle of 90° (normal to analyzer); the vacuum condition in the chamber was 2 × 10^–9^ Torr. High-resolution XPS spectra were measured with a pass energy of 20 and a 0.1 eV step size. The binding energies were calibrated using the C 1s peak energy as 285.0 eV. Data were collected and analyzed by using the ESCApe processing program (Kratos Analytical Ltd.) and Casa XPS (Casa Software Ltd.).

### 2.3 Electrode Preparation

The composite electrodes were prepared via a slurry coating method. The slurry was composed of 52.37 wt % of MCMB active material (pristine/coated), 0.57 wt % sodium carboxymethyl cellulose (Na-CMC, CRT 100, Walocel, Germany), 1.35 wt % styrene butadiene rubber (SBR, PSBR100, Targray, Canada), 0.57 wt % conductive agent (Super P Li, Timcal, Switzerland), 42.45 wt % deionized water (18 MΩ cm), and 2.70 wt % ethanol (p.a. grade, Merck). The slurry was homogenized by dispersing at a speed of 30,000 rpm for 1 h and finally cast on a smooth copper foil (Nippon foil, 10 μm thickness) using the doctor blade technique. The blade gap was set to 180 μm, and the resulting coating was dried at 80 °C under a reduced pressure of 0.005 mbar for at least 12 h. The as-coated electrodes had a thickness of ∼90 μm and a porosity of ∼50%. After calendaring, the thickness and the porosity were reduced to ∼30 μm and ∼30%, respectively. The areal weight loading of active material (MCMB or MCMB/MLD) for each dried composite electrode was ∼7 mg/cm^2^, which approximately corresponds to an areal capacity value of 2.5 mAh/cm^2^. Finally, circular electrodes with a diameter of 1.2 cm were stamped and used further in electrochemical measurements. On the other hand, both the MW-CNT tissue and the as-prepared MW-CNTs/MLD composite were used without any additional treatment in the form of freestanding circular electrodes with a diameter of 1.0 cm and a weight loading of ∼0.3 mg/cm^2^ stamped out from the original/modified tissue sample.

For full-cell testing, NCM composite electrodes were manufactured. In this case, the slurry was composed of 39.7% NCM_622_ (BASF), 1.9% conductive agent (Super P Li, IMERYS), 1.8% polyvinylidene fluoride (PVDF, Solef 5130 Solvay), and 56.6% *N*-methyl-2-pyrrolidone (Sigma-Aldrich Co.). The slurry was cast on a 16 μm Al current collector (99.3% purity, MIT Corp.) with a blade gap of 300 μm. Subsequently, the coating was dried under a fume hood for an hour, and the drying procedure was finished at 80 °C under a reduced pressure of 0.005 mbar for at least 12 h. Finally, the electrodes were calendared to approximately 35% porosity. The areal weight loading of active material (NCM_622_) for each dried composite electrode was ∼11.5 mg/cm^2^, which approximately corresponds to an areal capacity value of 2.0 mAh/cm^2^. Circular electrodes with a diameter of 1.0 cm were punched out and used for full cell cycling with an n/p ratio of ∼1.25.

### 2.4 Electrochemical Characterization

For the electrochemical characterization, the composite slurry-coated electrodes (MCMB, MCMB/MLD) as well as the as-prepared freestanding electrodes (MW-CNTs, MW-CNTs/MLD) were consistently assembled into the CR2032 coin cells (Hohsen Corp., two-electrode measurements) or PAT-cells (EL-CELL, three-electrode measurements) in a high-purity (99.999%) Ar-filled glovebox (MBraun, Germany) under a desiccated and deaerated atmosphere (H_2_O < 0.1 ppm and O_2_ < 0.1 ppm). A sheet of Li metal (99.9%, trace metal basis, MTI Corp.) was used as both the counter electrode (in half-cells) and the reference electrode (in three-electrode measurements), while in the case of full cells, we used the NCM_622_ cathodes. For coin cells, two layers of borosilicate-microfiber filter (0.7 μM pore size, Whatman) were used as a separator, while for PAT-cells, an FS-5P (EL-CELL) commercial separator was implemented. All cells were filled with an LP-30 electrolyte (battery grade, Sigma-Aldrich), containing 1 M lithium hexafluorophosphate (LiPF_6_) in ethylene carbonate (EC) and dimethyl carbonate (DMC), EC/DMC = 1:1 (v/v).

All electrochemical measurements were performed at room temperature. All potentials were measured and presented versus the Li^+^/Li reference electrode. Cyclic voltammetry (CV) measurements were conducted in a potential range of 0.01–3.00 V at a scan rate of 20 μV/s using a VSP-3e potentiostat (BioLogic). Galvanostatic charge–discharge measurements of the cells with MCMB (pristine/coated) electrodes were performed using a BCS-800 battery cycler (BioLogic), while those with MW-CNT (pristine/coated) electrodes were carried out using a BT2000 battery cycler (Arbin Instruments). Electrochemical impedance spectroscopy (EIS) measurements were performed in a frequency range of 10^–2^–10^5^ Hz at the respective open circuit potential with a peak-to-peak amplitude of 10 mV using a VMP3 potentiostat (BioLogic). The obtained Nyquist plots were fitted to the equivalent circuit^34^ using the ZView software (Scribner Associates Inc.). The exchange current density values were calculated from the obtained EIS spectra.^35^ In order to evaluate the Li-ion diffusion coefficient, the galvanostatic intermittent titration technique (GITT) experiments and subsequent calculations^36^ were performed with the MCMB (pristine/coated) electrodes using a VMP3 potentiostat (BioLogic).

## 3 Results and Discussion

### 3.1 Fabrication of Multiproperty Art-SEI via a Thermal MLD Technique

Considering the use of LP-30 commercial LIBs’ electrolyte (1 M LiPF_6_ dissolved in ethylene carbonate and dimethyl carbonate, EC/DMC = 50:50 (v/v)), as a genuine and yet quite complex MLD-precursor, the selection of an appropriate MLD coreactant(s) (cross-linking agent) as well as the adjustment of the manufacturing conditions (reactor temperature and pressure, pulse duration, etc.) were of particular importance. Among the variety of possible coreactants, we have chosen trimethylaluminum (TMA), which is a strong Lewis acid, holding a high reactivity toward nucleophilic functional groups/atoms.^37^ Such nucleophilic atoms, e.g., oxygen atoms at a double bond, are present in abundance in both EC and DMC. These two organic solvents constitute together about 88.3 wt % of the LP-30 LIBs’ electrolyte. Moreover, we have selected the TMA cross-linker to be able to bind the hexafluorophosphate (PF_6_^–^) anion, which is also classified as a weak nucleophilic agent.^38^ On the other hand, TMA cannot capture the solvated Li-ions, which are also present in the LP-30. In this situation, only covalently bound Li-ions, existing in the form of organolithium compounds, which must have a nucleophilic portion, can serve as an appropriate MLD precursor. Therefore, to realize the (pre)­lithiation of the Art-SEI, we introduced another MLD-precursor, namely, lithium *tert*-butoxide (LiO^*t*^Bu), which was applied after the first purging stage of the MLD-cycle protocol (see more details in the Experimental Section). The choice of LiO^*t*^Bu over other organolithium compounds was determined by its properties, i.e., relatively high vapor pressure at increased temperature (>150 °C) and good thermal stability.^39,40^ Furthermore, such a combination of the LiO^*t*^Bu precursor and the TMA coreactant has a strong presence in the ALD/MLD-fabricated thin-film coatings, despite the fact that these compounds do not represent a classical electrophile–nucleophile pair.^41–44^ Indeed, although LiO^*t*^Bu is generally classified as a Lewis base, its nucleophilicity is relatively weak due to steric hindrances at the central carbon atom.^40^

The choice and optimization of the temperature in the MLD reactor and in the precursors’ compartments were determined taking into account a number of considerations. We sought to intensify the evaporation of low-volatile precursor (LiO^*t*^Bu) and thereby ensure a rapid distribution of its vapors into the reactor chamber and, at the same time, prevent its premature thermal decomposition.^45^ The latter applies to all precursors used and could provoke the occurrence of several undesirable and competing reaction pathways. In addition, it is known that too high a temperature inside the MLD reactor slows down the adsorption of precursor molecules onto the substrate, as well as triggers the effect of dehydroxylation.^46^ Thus, the optimal reactor temperature was chosen to be 225 °C, which is close to the thermal stability limit of the two precursors, namely, LiO^*t*^Bu and DMC (a component in the LP-30).^40,47^ Moreover, higher reactor temperatures resulted in the formation of byproducts, such as aluminum fluoride (see Figure S2). The duration of each MLD pulse, as well as interpulse purges (see Table 1 in the Experimental Section), was optimized to ensure a sufficient mass transport of precursor molecules to the surface of each individual MCMB graphite particle and the complete removal of unreacted precursors and reaction byproducts after each reaction step.

While using the MCMB graphite as a model material, the effect of the number of MLD cycles on the electrochemical properties of the MLD-coated active material (MCMB/MLD) in a half-cell arrangement was studied (Figure S3). Specifically, the values of the initial capacity and Coulombic efficiency (irreversible capacity loss) in the first galvanostatic cycle were compared for the MCMB/MLD composite electrodes fabricated from the MCMB/MLD graphite powder obtained using different numbers (20, 40, and 80) of the MLD cycles. On the one hand, it is obvious that the application of 80 MLD cycles decreases both the charge and discharge initial capacities significantly, as compared with the uncoated (pristine) MCMB composite electrode. Also, the increased capacity observed during the lithiation curve (half-cell discharge) at the potential related to pristine SEI formation possibly indicates an irreversible process, involving the electroreduction of electrolyte components onto the thick MLD coating itself. This may well explain the decline in the electrochemical performance (in half-cell) of the electrode fabricated from the graphite powder experiencing 80 MLD cycles: the presence of a too thick layer (composed of the Art-SEI and on top of it the electrochemical “natural” SEI) may hinder the transport of Li-ions. On the other hand, the use of a smaller number of MLD cycles (20 and 40) significantly improves the Coulombic efficiency recorded for the electrodes and reduces the irreversible capacity loss associated with the formation of the SEI layer compared to the uncoated (pristine) MCMB composite electrode. In particular, the overall improvement of these parameters is enhanced once 40 MLD cycles are applied. As will be shown below, this effect is even more significant in the case of applying the MLD coating to the MW-CNT tissue.

### 3.2 Application of Art-SEI in MW-CNT Tissue Material as a Model System Possessing an Extensive SEI Formation

The cyclic voltammetry (CV) curve recorded for the first cycle in the polarization of a pristine and freestanding uncoated (reference) MW-CNT electrodes in the half-cell (Figure 1a, black curve) shows a pronounced cathodic peak at 0.7 V (vs Li/Li^+^), which is in good agreement with the literature and corresponds to the irreversible process related to the SEI formation via electrolyte components reduction and decomposition.^48^ It is well known that such a process irreversibly consumes Li-ions from the electrolyte (being compensated by an equivalent Li-ion consumption and loss from the Li metal counter electrode), converting them into insoluble compounds, such as LiF, Li_2_CO_3,_ and alkyl-carbonates, which do not contribute or participate directly in the cell charge–discharge.^49^

**Figure 1:**
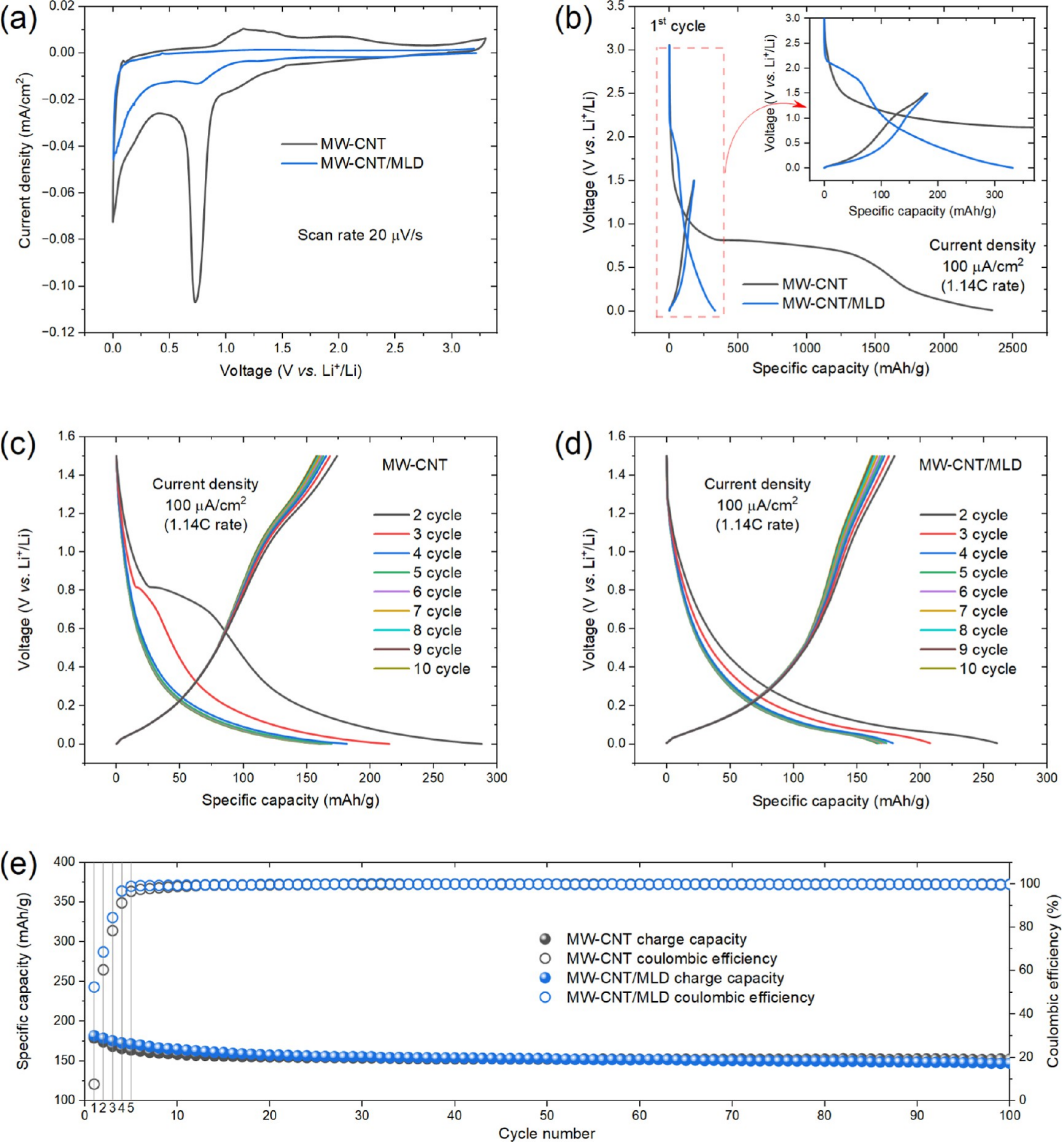
Cycling voltammograms at a scan rate of 20 μV/s (a), galvanostatic charge–discharge profiles at a current density of 100 μA/cm^2^ (1.14 C rate) for the 1st cycle (b) and for the 2nd–10th cycles (c,d), charge capacity and CE for 100 cycles (e), recorded for the MW-CNTs and MW-CNTs/MLD anodes in a half-cell configuration.

The generally accepted term for such irreversibly bound Li-ions is “dead lithium”.^50^ It is remarkable that in the case of a modified freestanding MW-CNT electrode coated with the implementation of an Art-SEI, the electroreduction process at 0.7 V is mostly mitigated (Figure 1a, blue curve). This means that the Art-SEI is truly functioning as an electrically insulating barrier layer, and at the same time, it is permeable for Li-ion migration and diffusion. Yet, the moderate cathodic current recorded at 0.7 V for the modified MW-CNTs/MLD electrode is indicative of an additional growth of a film over the established Art-SEI layer, where it is most probably insufficiently electrically insulating and/or nonconformal over the entire electrode surface. However, based on the relatively low cathodic peak amplitude in the case of the modified MW-CNTs/MLD electrode, one can assume almost a full substitution of the natural electrolytically formed SEI with the Art-SEI, which in turn fulfills the main SEI requirements, albeit now without the need for an extremely high irreversible reduction and decomposition of the electrolyte components in the first formation cycle.

Also, noteworthy is the fact that such a significant reduction in the irreversible capacity of the modified MW-CNTs resolves the main challenge, hindering the use of CNTs as freestanding anodes in flexible Li-ion batteries.^51,52^ Upon reversing the sweep into the anodic one (oxidation step), one can observe that the pristine (uncoated) MW-CNT electrode is experiencing an extended oxidation step, initiated at 0.9 V and continuing up to 2.5 V (vs Li/Li^+^). This behavior is not observed in the case of the modified MW-CNT electrode, possessing the Art-SEI originated from the novel MLD coating, and this serves as an excellent indication of the stability of the Art-SEI upon reversing the potential to a mild oxidative one. We also observe lower current density values recorded for the MLD-coated versus uncoated electrodes throughout the entire relevant range of applied reduction potentials (below 2.5 V and down to 20 mV vs Li/Li^+^), and this behavior may also result from the overall decreased surface area of the modified MW-CNTs/MLD electrode exposed to the electrolyte, and this aspect will be discussed in-depth later on.

A comparison of the constant current (galvanostatic) charge–discharge profiles of the first cycle recorded for the MLD-coated and pristine (reference) MW-CNT electrodes (Figure 1b) indicates a dramatic drop of the irreversible discharge (lithiation) capacity consumed within the SEI’s formation potential range (of <0.9 V vs Li/Li^+^).^8^ At the same time, the charge capacity, related only to Li-ion extraction (deintercalation) from the MW-CNTs, was found to be at the same level for both electrodes. Specifically, the irreversible capacity loss in the first cycle was mitigated from 583% for the uncoated electrode to 44% for the MLD-coated electrode, which in turn corresponds to the improvement in CE from 7.6 to 52.4%, respectively. An additional interest is the minor potential sloppy profile (elongated potential plateau) within the potential range of 2.1–0.7 V (vs Li/Li^+^), indicating a capacity loss (of ∼137 mAh/g) due to a modest electroreduction process recorded in this potential range (Figure 1b). As stated earlier, this relatively minimal capacity consumption may be related to an additional SEI growth proceeding, most likely within the depth of the MW-CNT tissue, completing the overall SEI formation, as indicated in the CV (Figure 1a) of the MW-CNTs/MLD electrode. This aspect will be discussed once we examine the surface morphology developed both on top and within the depth of MW-CNT tissue layers. In addition, Figure 1c,d shows the remarkable difference in the discharge–charge profiles between the two substances: while the pristine MW-CNT electrode needs an additional 2 cycles to establish a complete SEI buildup (evident by the second and third cycle potential plateaus at 0.825–0.65 V vs Li/Li^+^), the MW-CNT/MLD electrode does not show such behavior and actually the insertion of Li-ions occurred at no additional Li-ion consumption (irreversible capacity). Only from the fourth cycle onward do the two electrodes present similar behavior, as manifested in Figure 1e, presenting the cycling performances and Coulombic efficiencies of the two electrodes in the half-cell configuration.

Next, we obtained the first-cycle impedance spectra for the pristine and MLD-modified freestanding MW-CNT electrodes in half-cells (Figure 2a). These spectra were analyzed using the mechanistic approach of curve fitting to an equivalent circuit.^53^ The concrete equivalent circuit (presented as an inset in Figure 2b) for such analysis was chosen based on the literature data.^34^ According to this circuit, the overall resistance is composed of three components associated with the Ohmic losses (*R*_e_), SEI film (*R*_SEI_), and Li-ion transfer (*R*_CT_). The impedance spectra for the first ten galvanostatic charge–discharge cycles (shown in Supporting Information Figure S4) were analyzed, each at the same state of lithiation (SoL) equal to 0.50. As a result, the dependence of the electrode resistance components on the cell cycle number was obtained (Figure 2b). Obviously, in the case of the pristine-uncoated (reference) MW-CNT tissue electrode, the *R*_SEI_ makes the greatest contribution to the total electrode resistance. Importantly, the presence of an Art-SEI essentially reduces the *R*_SEI_ value, making its contribution to the overall resistance much less significant. Specifically, over all the first 10 cycles, the *R*_SEI_ was recorded to be at essentially lower values (5–10 Ω) in the case of the MLD-coated MW-CNT electrode, compared with that of the pristine-uncoated MW-CNT electrode (18–35 Ω).

**Figure 2:**
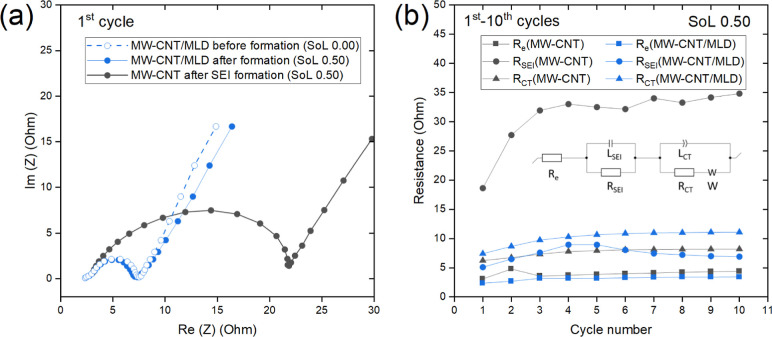
(a) First-cycle electrochemical impedance spectroscopy (EIS) profiles of pristine and MLD-coated MW-CNT electrodes recorded before and after SEI formation at 0.00 and 0.50 states of lithiation (SoL), respectively. (b) Impact of the different components (*R*_e_, *R*_SEI_, *R*_ct_) on the overall interfacial resistance of pristine/coated MW-CNT electrodes obtained by a curve fitting of the impedance spectra using the equivalent circuit^34^ model (presented as an inset) for the first 10 charge–discharge cycles. Plotting these components versus cycle numbers demonstrates their evolution during cell cycling.

The surface morphologies of the pristine and MLD-coated MW-CNT freestanding tissue electrode materials were studied by using SEM analysis (Figure 3a,c,d). It was found that the MLD coating on the MW-CNT tissue sample is nonuniform. Specifically, while on top of the MW-CNT tissue sample, a thick conformal MLD coating is observed (Figure 3c), an in-depth (inner layer) inspection of the modified tissue reveals a near absence of evidence of a noticeable and visible coating (Figure 3d). We attribute such inhomogeneous deposition to a slow diffusion of MLD-precursors through the densely packed layers constituting the MW-CNTs (Figure 3b). Quantitatively, the rate limitation of the diffusion of gaseous MLD-precursor molecules through the MW-CNT tissue in the MLD reactor can be roughly estimated based on literature data on the synthesis of the closed-packed CNT arrays via chemical vapor deposition (CVD), where the same flow rate (60–100 sccm) of carrier gas was applied.^54^ In this process, carbon deposition is determined and controlled by diffusion of the precursor molecules through the channels and pores present in the close-packed CNT array, thus limiting the growth rate, which is only about 0.9–1.0 Å/min. Such slow growth of the deposit thickness due to the slow diffusion of gaseous precursor molecules through the nanotube array is a good demonstration of the observed phenomenon. Thus, considering the time of precursor exposure in each MLD cycle (11.5 s) and the number of the applied cycles (40), one can expect the deposition of a coating thickness of no more than ∼0.8 nm, which is far below the detection and resolution limits of a SEM device. Moreover, it has been demonstrated that after a certain thickness of a deposit on the CNT walls is reached, depending on the conditions of such synthesis, the growth rate slows down due to the reduction in the size of the channels and pores through which the precursor molecules diffuse through the CNT array.^54^ Further passage of the precursor through the synthesis reactor leads to the deposition of carbon largely above the CNT array, rather than in the inner lumen of the pores, leading initially to the formation of closed pores and then a continuous bulk layer of carbon depositing above the entire CNT array sample. This is exactly what is observed in the current work being reported: a continuous coating over the entire MW-CNT sample and the absence of a (hard-to-detect) coating in the core of the MW-CNT tissue (Figure 3d).

**Figure 3:**
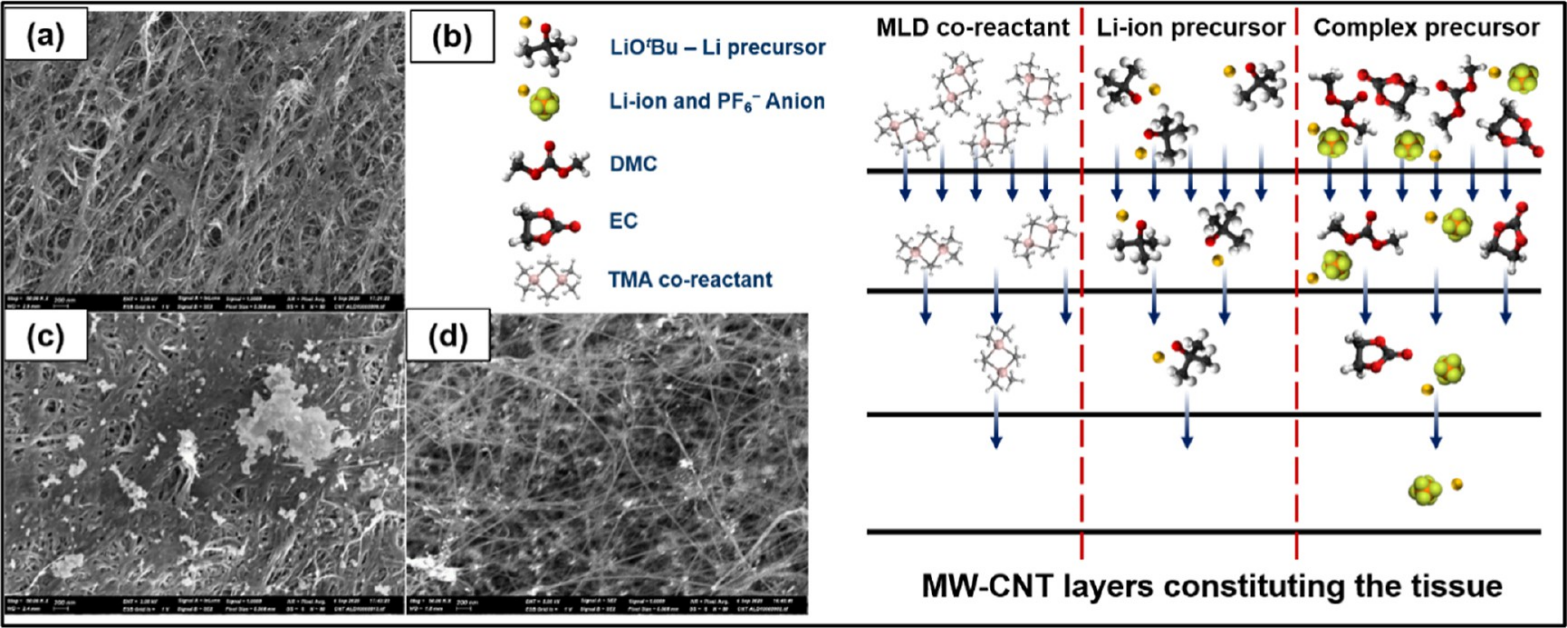
SEM images of pristine (a) and MLD-coated (c,d) MW-CNT tissue samples. The permeability of MW-CNT tissues for the MLD-precursor molecules is lower for big molecules and higher for small molecules, which results in the nonhomogeneous distribution of MLD coating in-depth of MW-CNT sample, i.e., on top of MW-CNT tissues—a large amount of the deposited coating was observed (c), while within MW-CNT inner layers, only a small amount of the coating was detected (d). The scheme (b) demonstrates the transport of various MLD-precursor molecules to the depth of MW-CNT tissues.

We can assume at this stage that the minor irreversible capacity still observed for the modified MW-CNTs with an Art-SEI and discussed earlier (Figure 1 and related discussion) may originate from a partial exposure of the MW-CNTs at the subsurfaces to the electrolyte due to an incomplete and/or nonuniform MLD coverage of the Art-SEI within the MW-CNT inner layers. This challenge can be overcome by implementing the novel MLD processing in a fluidized bed reactor type, aiming at the development of an Art-SEI on powder materials. Therefore, in the second part of this work, we aimed our research at a commercial powdered active anode material, namely, MCMB, as a substrate for molecular engineering of an Art-SEI on the surface of each individual graphite microparticle.

### 3.3 Application to a Commercial Anode Material

The same MLD protocol was applied to an MCMB graphite powdered material but now using a fluidized bed reactor type (see Experimental Section). The SEM images comparing the pristine and MLD-coated MCMB graphite particle monolayer arrays supported on an adhesive copper tape (Figure 4a,b) reveal no visible difference in surface morphology. To reveal the elemental composition and molecular “fingerprints”, as well as to establish the concrete bonds forming in the applied MLD-coating process, the additional spectroscopic and microscopic measurements were accomplished and analyzed.

**Figure 4:**
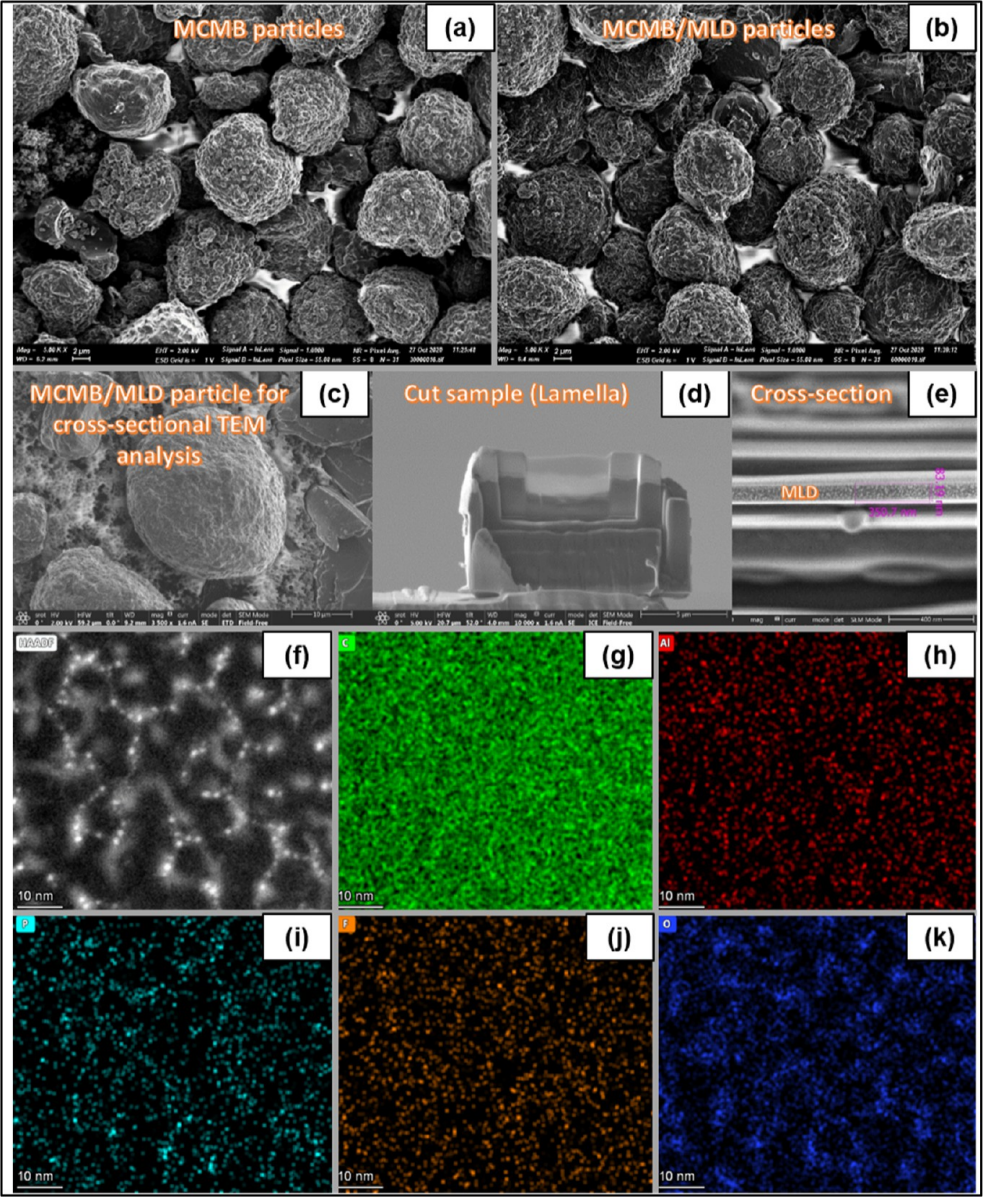
SEM images of the pristine (a) and MLD-coated (b) MCMB particle arrays. FIB-SEM images of one individual MCMB/MLD microparticle chosen for the cross-sectional TEM analysis (c), lamella sample cut from it (d), and MLD-film cross section (e). TEM structural and elemental cross-sectional analysis: the HAADF-STEM images (f) and corresponding STEM-EDS single-element mapping images (g–k) of the MLD-film cross section recorded for the different elements: C (g), Al (h), P (i), F (j), and O (k).

Accordingly, the FTIR spectrum (Figure S5) has been recorded from the surface of the MCMB/MLD graphite powder taken from the same batch that the microparticles presented in Figure 4b. Comparison of this FTIR spectrum with those published previously by Aurbach and co-workers^55^ obtained from the surface of negative electrodes either in situ upon Li-ion cell functioning or ex situ when the electrodes were extracted from Li-ion cells filled with a similar electrolyte composition after the cycling process, and thus having a layer of natural SEI on their surface, allows us to draw important conclusions.

First, it is worth noting the presence of key markers, indicating the presence of the main components of a natural SEI, i.e., Li-carbonates and Li-alkyl carbonates in the composition of the Art-SEI. Specifically, peaks at 1640 (υ_C=O_, as.), 1402 (δ_CH_2__), 1307 (υ_C=O_, s.), 1075 (υ_C–O_), and 838 ( $\delta_{{-OCO}_{2}^{-}}$ ) cm^–1^ reliably identify the presence of lithium ethylene dicarbonate ([CH_2_OCO_2_Li]_2_).^56^ The simultaneous appearance of the peaks at 2877 (υ_CH_), 1640 (υ_C=O_, as.), and 1329 (υ_OCO_2__) cm^–1^ indicates the presence of lithium methyl carbonate (CH_3_OCO_2_Li).^55^ Also, peaks at 1516 and 838 ( $\delta_{{-OCO}_{2}^{-}}$ ) cm^–1^ are indicative of the presence of lithium carbonate (Li_2_CO_3_).^55,57^

It should be noted that there are two metal cations in the designed Art-SEI layer, namely, lithium and aluminum cations. Thus, possibly both cations may be present in the composition of the carbonates under consideration. Indeed, as will be discussed and presented below, from the X-ray photoelectron spectroscopy (XPS) measurements and analysis, the total content (atomic concentration) of aluminum in the Art-SEI layer is 1.87 times greater than that of lithium and the detected aluminum can be in both the Al–C and Al–O forms. Accordingly, both Li-ions and Al-ions are considered to be the cations of the detected carbonates in the Art-SEI layer; this by itself portrays the composition of the Art-SEI fundamentally different from the natural SEI layer. The fact that the molecular composition of the Art-SEI layer developed in this work is different from the composition of the natural SEI layer was also evidenced by the presence of additional peaks at 974, 1182, 1284, and 1770 cm^–1^ in the FTIR spectrum of the Art-SEI layer. We attribute the first two peaks (974, 1182 cm^–1^) to the presence of Al–O and Al–C bonds, respectively.^58^ As an additional point, the absence of LiF-related peaks in the FTIR spectrum is noted: LiF is among the commonly presented constituents of natural SEI, originating from the reduction of the LiPF_6_ salt;^55^ albeit in this work, it is not present in the formed Art-SEI film, and one may regard this as a distinctive characteristic of the formed Art-SEI layer.

It is quite difficult to establish a specific mechanism of interaction of the nucleophilic groups presented in EC, DMC, and LiO^*t*^Bu molecules as well as of PF_6_^–^ anions with a TMA strong electrophile. Obviously, here TMA acts as a chemical reducing agent. However, such interaction seems to be quite complex, by analogy with the already studied electrochemical reduction of EC and DMC molecules that occurred in a Li-ion cell, which includes processes such as ring-open reduction (EC only), formation of radicals, their transformation, and subsequent reaction with each other.^59,60^ Thus, the study of all stages and intermediate products of the MLD process should be the subject of a future detailed study including in situ spectroscopic measurements as well as electrogravimetric (quartz crystal microbalance) analyses. Nevertheless, the molecular composition of the obtained MLD coating was additionally studied by the ex situ XPS method.

XPS measurements (Figure 5 and Table 2) were obtained and recorded from the surface of the MCMB/MLD graphite powder (taken from the same batch of the microparticles presented in Figure 4b). Qualitative and quantitative analyses of these XPS spectra show the strong presence of C–C, C–O, C=O, and CH bonds (Figure 5a,b) constituting the Li­(Al)-carbonates and Li­(Al)-alkyl carbonate species, which were detected earlier in the FTIR measurements (Figure S5). Of particular interest is the XPS spectrum of Al 2p (Figure 5c), showing that aluminum, which is present in the artificial layer in an amount of 12.99 ± 0.06 at. % (Table 2), acts as a cross-linker, connecting alkyl carbonate molecules predominantly through Al–O bonds. As will be shown further in Section 3.5, such aluminum-cross-linked alkyl carbonates exhibit high resistance against oxidation/hydration upon long-term exposure to an open environment (air and humidity), thus maintaining the essential electron-barrier and ion-transport properties.

**Figure 5:**
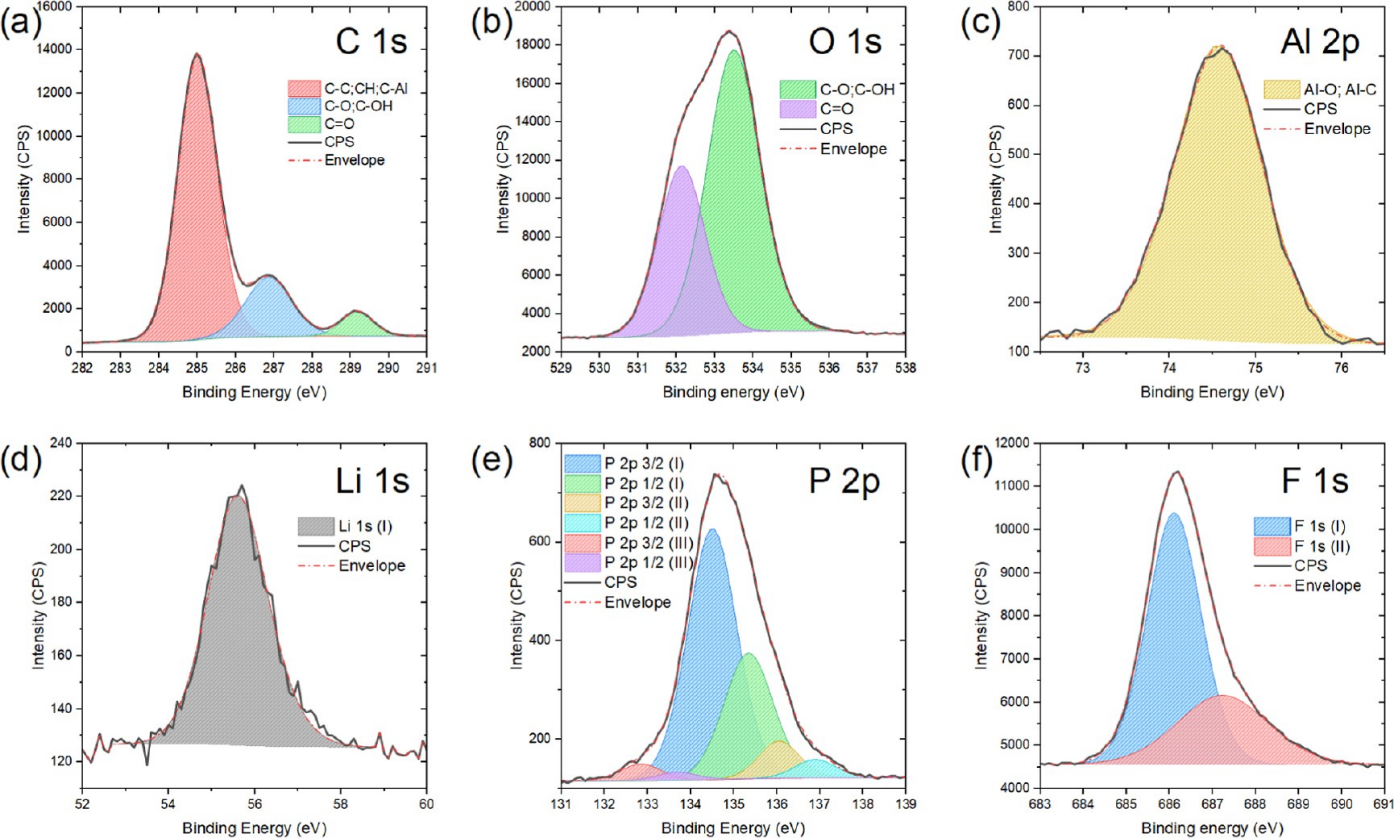
XPS spectra of C 1s (a), O 1s (b), Al 2p (c), Li 1s (d), P 2p (e), and F 1s (f) regions of the MCMB/MLD graphite powder surface obtained on the same array of MCMB/MLD microparticles presented in Figure 4b.

**Table 2:** A Quantitative XPS Analysis of MCMB/MLD Microparticles Presented in Figure 4b^a^

element	C 1s	O 1s	Al 2p	Li 1s	P 2p	F 1s
atomic conc., %	40.58 ± 0.23	26.87 ± 0.19	12.99 ± 0.06	8.36 ± 0.16	1.59 ± 0.12	9.59 ± 0.15
mass conc., %	31.29 ± 0.25	27.60 ± 0.22	22.51 ± 0.10	3.73 ± 0.08	3.17 ± 0.25	11.70 ± 0.20

a Relative atomic and mass contents of the elements calculated as average values from the XPS depth profiling (Figure S6).

In addition, the presence of significant amounts of P 2p and F 1s (Figure 5e,f) indicates that the PF_6_^–^ anions present in the MLD complex precursor are adsorbed electrostatically on the surface of the growing MLD film and are gradually immured in it, thus providing a sufficient level of its nucleophilicity for better subsequent cross-linking by the TMA electrophilic agent. Finally, the presence in the Art-SEI of Li 1s (Figure 5d) in a relative amount of 8.36 ± 0.16 at. % (Table 2) is much lower than that in the electrolytically formed natural SEI.^61^ This finding is very important because it demonstrates that the functioning of Li-alkyl carbonate-based interphases in LIBs’ anodes can be possibly realized even at significantly lower Li-ion contents.

In order to evaluate the inner structure of the produced MLD coating, as well as to verify the presence of the main molecular constituents of Art-SEI in its composition, we performed the cross-sectional structural and elemental mapping analysis using focused ion beam scanning electron microscopy (FIB-SEM) and transmission electron microscopy (TEM) methods. Specifically, the MCMB/MLD particles array was coated with a protective mask (Pt–C) using an ion-deposition technique, followed by the lamella-sample (Figure 4d) preparation from the surface of one individual MCMB microparticle (Figure 4c) by means of FIB-SEM microscope. The FIB-SEM image of the MLD-film cross section (Figure 4e) proves the formation of a conformal MLD film, ca. 83 nm in thickness. Such a large thickness of the MLD coating implies a complex interaction between TMA, DMC, EC, PF_6_^–^, and LiO^*t*^Bu molecules and anions with the surface of the growing MLD film, resulting eventually in a ca. 2.08 nm/cycle effective growth rate. The high-angle annular dark-field (HAADF) scanning-TEM (STEM) image of the MLD cross section (Figure 4f) shows a porous scaffolding structure permeated with a large number of nanosized (5–10 nm) voids. The presence of such large voids in the MLD-coating structure could be an additional rationale for this atypically rapid effective growth per cycle rate (2.08 nm/cycle). Importantly, the uniform distribution along the coating depth and in the lateral direction of all the elements (C, Al, P, F, and O) constituting the MLD film, which should be said to have already been quantitatively confirmed by the XPS measurements (Figure 5, Table 2). In particular, the STEM energy-dispersive X-ray spectroscopy (EDS) analysis (Figure 4g–k) proved that the MLD coating on the MCMB graphite particle is composed of both the organic and inorganic components of the LP-30 electrolyte as well as Al-based cross-linking molecules. As a final point, all applied cross-sectional analyses show that both the porous structure and the elemental composition of the MLD layer remain the same in whole depth within the MLD coating, thus proving a uniform (with no strata) buildup and identical deposition conditions from the very beginning to the very end of the MLD-coating process.

In addition, XPS depth profiling measurements were applied to the surface of the MCMB/MLD graphite powder (Figure S6), allowing reliable conclusions to be made on the distribution of MLD layer constituents within the coating depth. The MLD-coating composition remains stable up to a depth of approximately 70 nm, where the carbon content starts increasing and the content of all other components decreases, which indicates that the process of etching has reached the depth at which the substrate (MCMB graphite) is located. It is important to emphasize here that the XPS depth profiling was performed on a powder sample and not on a flat plate. Therefore, no clear boundary was observed between the MLD coating and the MCMB graphite substrate. In general, the XPS depth profiling results are in good agreement with the TEM cross-sectional analysis (Figure 4): namely, a uniform depth distribution of the MLD-coating components, such as Li­(Al)-alkyl carbonates, is confirmed.

The uniformity and conformality of the Art-SEI layer are critical parameters affecting its protective functionality. Thus, to validate the homogeneity and thickness uniformity of the Art-SEI layer, a cross-sectional study, including high-resolution FIB-SEM and/or TEM, is required. However, certain limitations were revealed for the MLD/MCMB cross-sectional interface (TEM lamella) preparation, resulting in a rather large overestimation of the coating thickness during FIB-SEM and TEM measurements. To demonstrate this phenomenon, an additional TEM analysis of a cross section of the MLD/MCMB interphase after the application of a protective mask was performed (Figure S7). Specifically, it was found that the application of a 90% Pt–10% C protective mask using directed ion deposition (see the Experimental Section) leads to the penetration of Pt-ions through the porous layer of the MLD film and even to some depth (up to 0.3 μm) of the MCMB graphite microparticle, which is also very porous (Figure S7d). Furthermore, and most importantly, the transfer (carrying over) of a portion of the atoms of the MLD-coating constituents together with the Pt-ions into the depth of the graphite microparticle was revealed, as well as upward in the direction of the protective mask, together with the platinum atoms that bounced off the solid graphite (Figure S7e–h). It should be noted that the reason for such an ablation of atoms along the cross section into the depth of the MCMB graphite and in other directions can be both the process of ion beam deposition of a 90% Pt–10% C protective mask and the subsequent FIB Ga-ion-beam milling process during the cross-sectional interface (TEM lamella) preparation (see the Experimental Section). It cannot also be ruled out that a small amount of MLD-precursor molecules is able to penetrate the upper porous MCMB graphite layer. As a result, the thickness of the MLD coating observed in the cross-sectional FIB-SEM or TEM images of the MLD/MCMB interface is greatly overestimated, and the magnitude of such overestimation can reach 250–300 nm. Despite the methodological difficulties described in the process of determining the exact value of the MLD coating’s thickness, the detailed analysis of Figure S7b indicates that this coating is continuous and its thickness is in the range of 75–90 nm.

A similar cross-sectional TEM comparative analysis, but now postmortem, has been applied to microsized graphite species extracted from the MCMB and MCMB/MLD composite electrodes that had been subjected to 100 charge–discharge cycles at a 0.1 C rate (shown in Supporting Information Figure S8). It is obvious that on the electrode made of a pristine MCMB graphite material, the SEI layer is continuous and has a thickness of 0.46–1.12 μm. In contrast, on the modified MCMB/MLD electrodes, the additional growth of the SEI layer is fragmentary, thus increasing the total SEI thickness only in some areas by no more than 0.28 μm. Thus, the developed MLD coating largely imitates the SEI layer, providing interphase transport of lithium ions and at the same time serving as a reliable barrier for electron transfer, thus greatly reducing the electrochemical decomposition of the lithium-ion electrolyte. The elemental analysis (Figure S8c–j) showed that the SEI layer on both electrodes consists of elements included in the electrolyte, which is reliable evidence that the identification of the SEI layer in the TEM images (Figure S8a,b) was correct.

In an effort to prove that it is the complex composition of the MLD coating based on the molecular chemistry of the Li-ion electrolyte and not the presence of oxidized aluminum in the MLD thin film, we carried out a similar TEM analysis of graphite specimen extracted from the MCMB/Al_2_O_3_ electrodes that had been subjected to 100 charge–discharge cycles at a 0.1 C rate (Figure S9). In this case, the modification of the MCMB graphite material with the Al_2_O_3_-ALD (40 ALD-cycles) coating was performed as described elsewhere.^26,27^ The SEI layer on the MCMB/Al_2_O_3_ electrode is continuous and has approximately the same thickness as the SEI layer on the MCMB electrode (Figure S8a); this is important evidence that the oxidized aluminum (Al_2_O_3_) layer is not able to provide the mitigation of the Li-ion electrolyte decomposition (SEI layer growth).

Next, the MLD-modified MCMB graphite powder was applied as an active material for the preparation of the composite anodes. It is important to note here that we used a state-of-the-art water-based binder, namely, sodium carboxymethyl cellulose/styrene butadiene rubber (Na-CMC/SBR), which is utilized in graphite-based anodes to maintain long-term cycling stability.^62^ In this regard, the stability of the electrochemical properties of the produced MLD coating during Li-ion cell cycling, and even more importantly, against an exposure to aqueous media (water-based binder processing) was verified versus the noncoated MCMB-based anodes (Figure 6).

**Figure 6:**
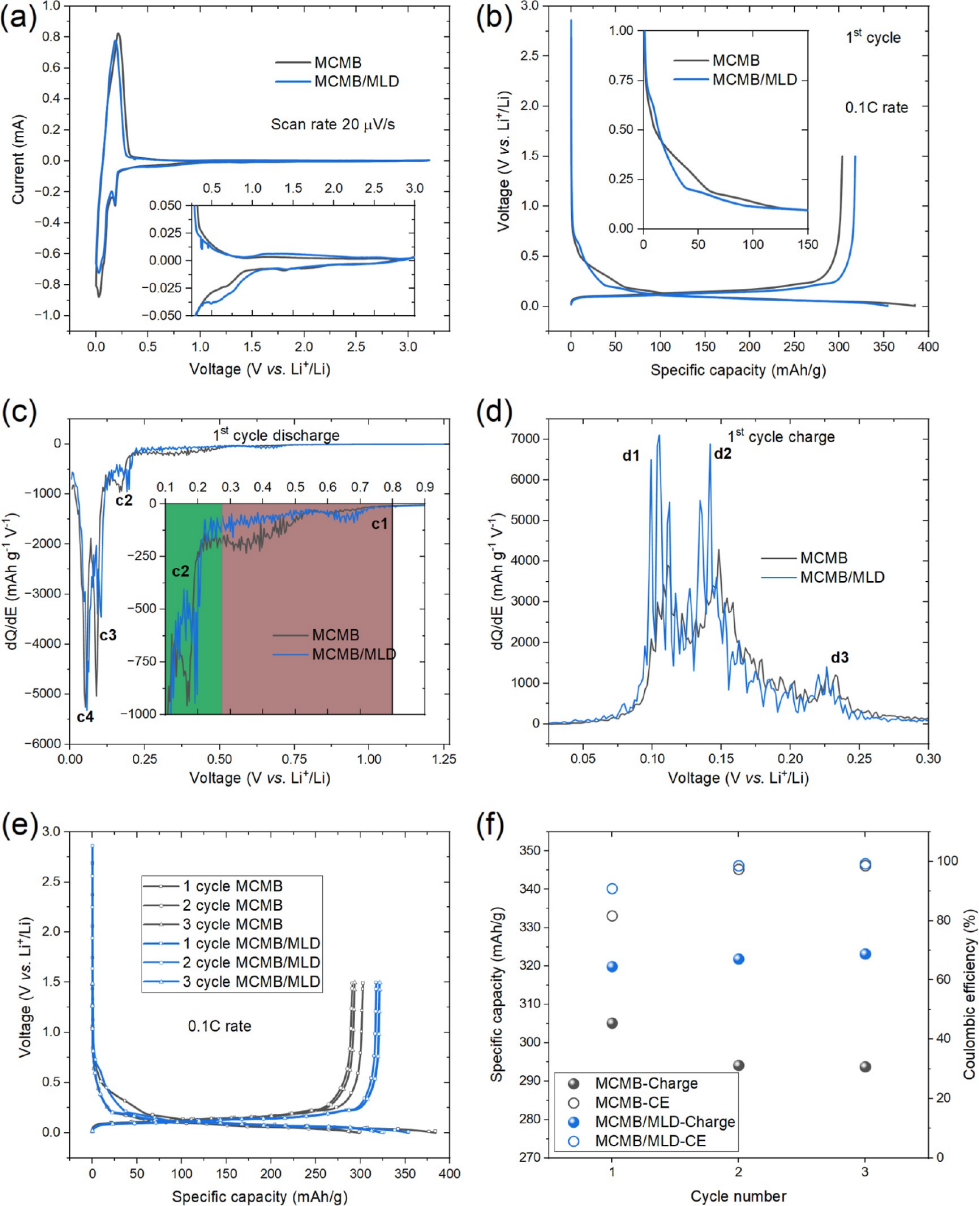
Cycling voltammograms at a scan rate of 20 μV/s (a), galvanostatic charge–discharge profiles of the first formation cycle at a 0.1 C rate (b), corresponding d*Q*/d*E* plots (c,d), galvanostatic charge–discharge profiles of the initial three cycles at a 0.1 C rate (e), charge capacity and CE for 3 cycles (f), recorded for the MCMB and MCMB/MLD anodes in a half-cell configuration. The d*Q*/d*E* peaks in (c,d) are labeled, and the assigned reactions are discussed in the text below. The potential regions typically associated with the SEI formation and the subsequent Li-ion intercalation into the graphite are highlighted with brown and green colors in (c).

Such verification is of special interest and significance, given the role of the applied MLD coating, i.e., to mimic and to consistently replace natural SEI with an artificial one, thereby immediately overcoming the inevitable loss of capacity in the initial (SEI formation) cycles (Scheme 1). In particular, such stability of the MLD coating against exposure to aqueous media could add an additional and huge value to this Art-SEI concept.

The performance of natural SEI is extremely sensitive to the presence of water even in trace amounts, promoting additional SEI growth (SEI thickening), increased impedance, and hydrolysis of lithium salts with subsequent formation of HF.^63^ If all of these undesired processes can be prevented by designing a robust and water-stable Art-SEI layer, it will be a breakthrough and a “giant leap” forward in the manufacturing of Li-ion batteries. Specifically, the CV measurements (Figure 6a) recorded in a half-cell configuration in a three-electrode cell configuration for the pristine (uncoated) and MLD-coated MCMB powders constituting the composite electrodes, revealed that the first intercalation/deintercalation process proceeds at more reversible conditions for the MCMB/MLD electrode.

Indeed, while the position of two cathodic (Li-ion intercalation) peaks remains unchanged, the position of the anodic (Li-ion deintercalation process)-related peaks shifts in a negative direction, indicating an easier extraction of the stored Li-ion, manifested by a lower anodic overpotential (of ∼50 mV) for Li-ion extraction from the Art-SEI-modified MCMB lithiated graphite material, compared with the regular MCMB-based electrode. In other words, Li-ion migration from the graphite lattice through the Art-SEI, back to the electrolyte, requires slightly less energy investment. This statement finds additional support when we inspect the constant current (galvanostatic) charge–discharge profiles recorded for the first cycle (Figure 6b) as well as from the corresponding differential capacity (d*Q*/d*E*) versus voltage plots recorded for the process of Li-ion intercalation (Figure 6c).

From Figure 6c, it is evident that less capacity typically associated with the SEI formation was recorded for the MCMB/MLD electrode, specifically for the potential region of 800 mV down to 275 mV (c1, brown), where the SEI growth occurs. In the subsequent potential region (c2, green), starting from 275 mV and downward, the Li-ion intercalation process starts into the graphite. Finally, well-defined peaks are recorded at 90 and 60 mV (c3 and c4) corresponding to the characteristic lithiation states of graphite, LiC_12_, and LiC_6_, respectively. Similarly, the d*Q*/d*E* plot obtained for the anodic process (Figure 6d) indicates faster lithium deintercalation at lower anodic overpotentials for the MCMB/MLD electrode compared to that for the MCMB electrode. This is evident from the intensities and shifts of the peaks d1, d2, and d3, which correspond to the characteristic deintercalation processes. Moreover, during the cell charging process, the reversibly accumulated in the MCMB/MLD electrode Li-ions are transferred back to the electrolyte with an essentially higher Coulombic efficiency (CE = 90.93) compared to that recorded for the pristine (uncoated) MCMB electrode (CE = 81.73) (Figure 6b). The difference between the number of Li-ions involved in the first intercalation process and those participating in the first deintercalation action constitutes the irreversible loss of capacity, which is much lower in the case of the MCMB/MLD electrode. Furthermore, the registered value of the overall electrode capacity was significantly higher and more stable in the initial cycles for the case of the MCMB/MLD electrode operating in half-cells (Figure 6e,f).

It is noted herein that the presence of a water-based binder processing in the composite electrode primary (before vacuum drying) formulation in all these experiments did not cause oxidation/hydration and, therefore, did not reduce the electrochemical performance of the Art-SEI layer. Specifically, no additional peaks were detected on the cyclic voltammograms (Figure 6a) or in the galvanostatic charge–discharge profiles (Figure 6b,e) that would indicate the interaction of artificial SEI with water or the electroreduction of the previously oxidized MLD layer. It is assumed that such chemical and electrochemical stability of the Art-SEI layer with respect to a water-based binder is explained by the use of a TMA cross-linker, which is supposed to firmly bind the functional groups of the organic precursor molecules, preventing the possibility of their subsequent oxidation/hydration in the presence of water.

To study the impact of Art-SEI on the electrode interfacial kinetics, the diffusion coefficient of Li-ions and the effective (per unit of weight) exchanged current density (analogous to the rate constant in chemical reactions) were evaluated and plotted versus the potential for the MCMB and MCMB/MLD electrodes in half-cells (Figure 7). These measurements were accomplished after SEI formation during the second discharge of the half-cell. Obviously, the Art-SEI is characterized by faster ionic diffusivity and higher exchange current density as compared with natural SEI. Specifically, in the potential range of 0.02–0.40 V, the diffusion coefficient of MCMB and MCMB/MLD electrodes turned out to be within the range of 10^–8^–10^–7^ cm^2^ s^–1^ and 10^–7^–10^–6^ cm^2^ s^–1^, respectively, with a few sharp disturbances appearing at potentials below 0.15 V. Likewise, the effective exchanged current density for MCMB and MCMB/MLD was found to be in the range of 0.10–0.15 and 0.17–0.23 A/g, respectively, besides a significant slowdown registered at the highest SoL level. We attribute the faster interfacial kinetics through Art-SEI over natural SEI to the highly porous structure (Figure 4f) of the Art-SEI layer, where the formation of Li-ion pathways requires less energy.^64^ In general, electrodes with low porosity also have increased tortuosity, which slows down the transport of Li-ions.^65^

**Figure 7:**
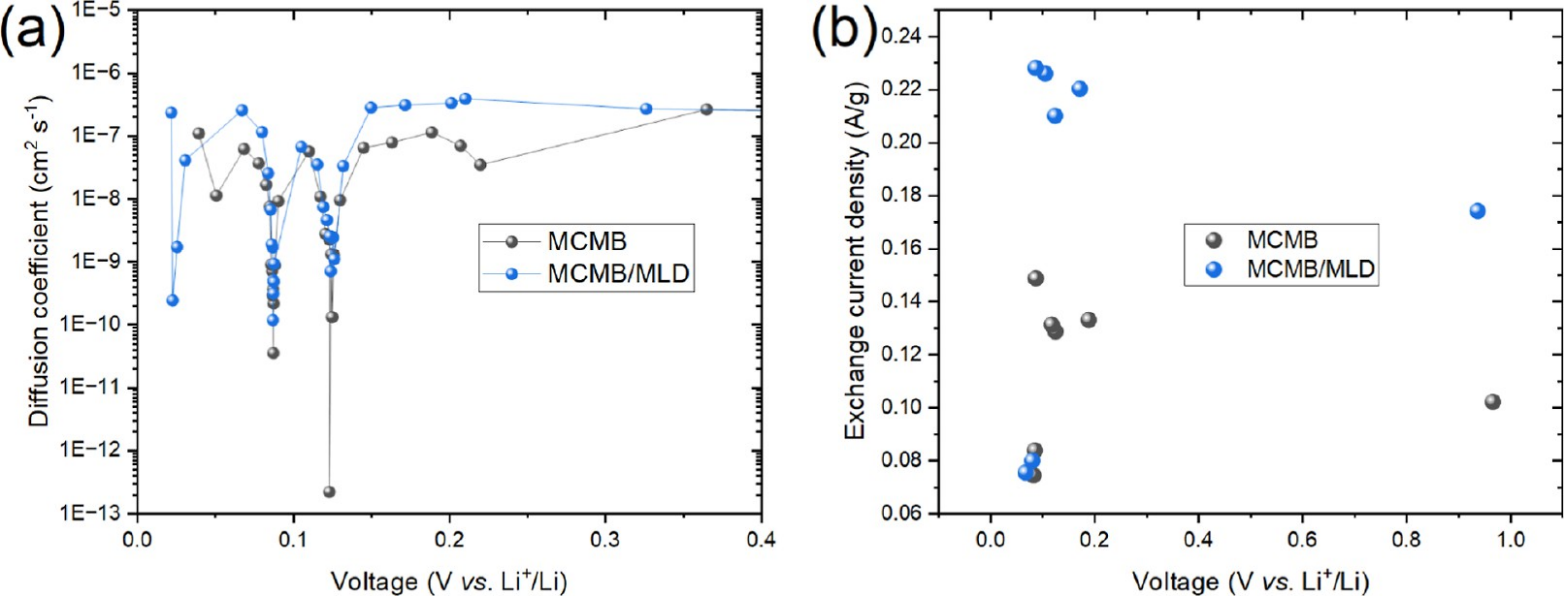
Diffusion coefficient of Li-ions versus voltage (a) and exchange current density versus voltage (b) plots recorded for uncoated MCMB and MCMB/MLD composite electrodes in a half-cell configuration.

The EIS analysis of the MLD-coated versus pristine MCMB graphite electrodes recorded in the first cycle at different states of lithiation (SoL) values, i.e., 0.03, 0.5, and 0.92, is presented in Figure 8a–c. As in the case of MW-CNT tissue electrodes, the Nyquist plots are composed of one or two semicircles at the high-frequency region, representing the resistance components associated with the combined charge transfer through the SEI film (*R*_SEI_) and through the electrode–electrolyte interface (*R*_CT_); and a sloping line at the low-frequency region related to the resistance associated with Li-ion diffusion in the bulk electrode (*R*_e_). It is obvious that the overall electrode resistance is lower in the case of the MLD-coated MCMB electrode (blue curve at Figure 8a–c) than for the pristine one (black curve at Figure 8a–c), especially at lower SOC values (0.03 and 0.5), which is achieved solely due to the gain (decrease) in *R*_SEI_ and *R*_CT_ values.

**Figure 8:**
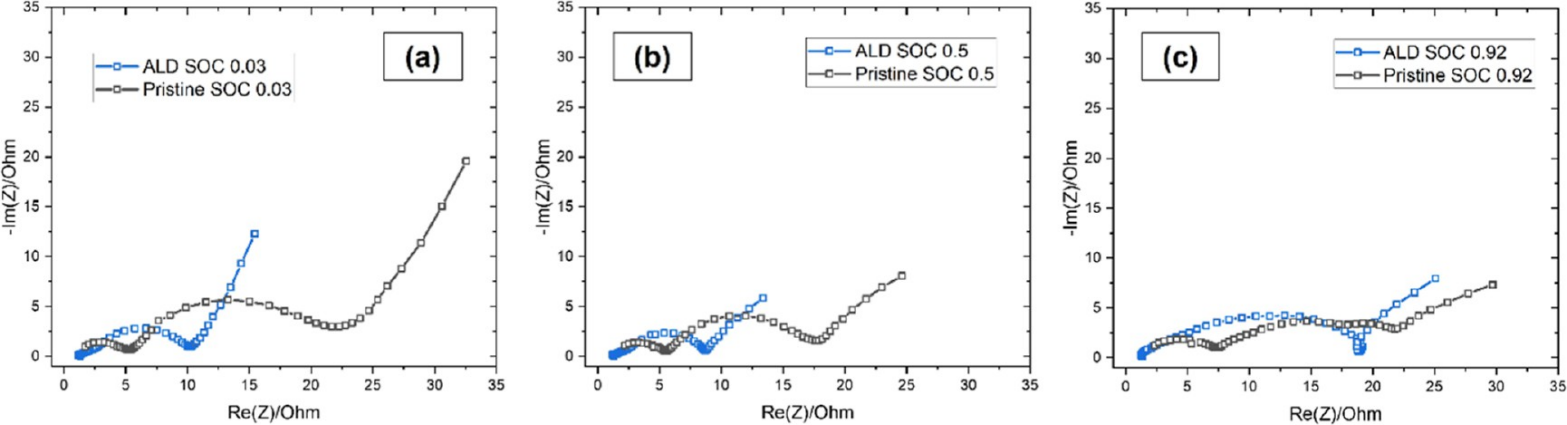
First-cycle EIS profiles of pristine and MLD-coated MCMB electrodes recorded at different states of lithiation (SoL): 0.03 (a), 0.50 (b), and 0.92 (c).

Moreover, the comparative cycling performance of the MCMB/MLD versus MCMB composite anodes was studied in a full-cell configuration with a commercial cathode active material (NCM_622_) in a three-electrode arrangement (Figure 9). Specifically, the cycling protocol consisted of the 3 cycles at a 0.1 C rate followed by 50 cycles at a 1 C rate. Such a protocol was repeated 20 times (1060 cycles total) to estimate the overall sustainability under the increased C-rate. It was established that the MCMB anodes did not withstand the 1 C-rate cycling demonstrating very low (<5 mAh/g) discharge capacity (Figure 9a,c), while the MCMB/MLD anodes showed much better capacity (>50 mAh/g), which has been even increasing steadily up to the 250th cycle (activation effect) and remained stable to the end of the test (Figure 9b,c). We attribute these results to the improved kinetics of Li-ion transport through the Art-SEI layer as compared with that through the natural SEI layer.

**Figure 9:**
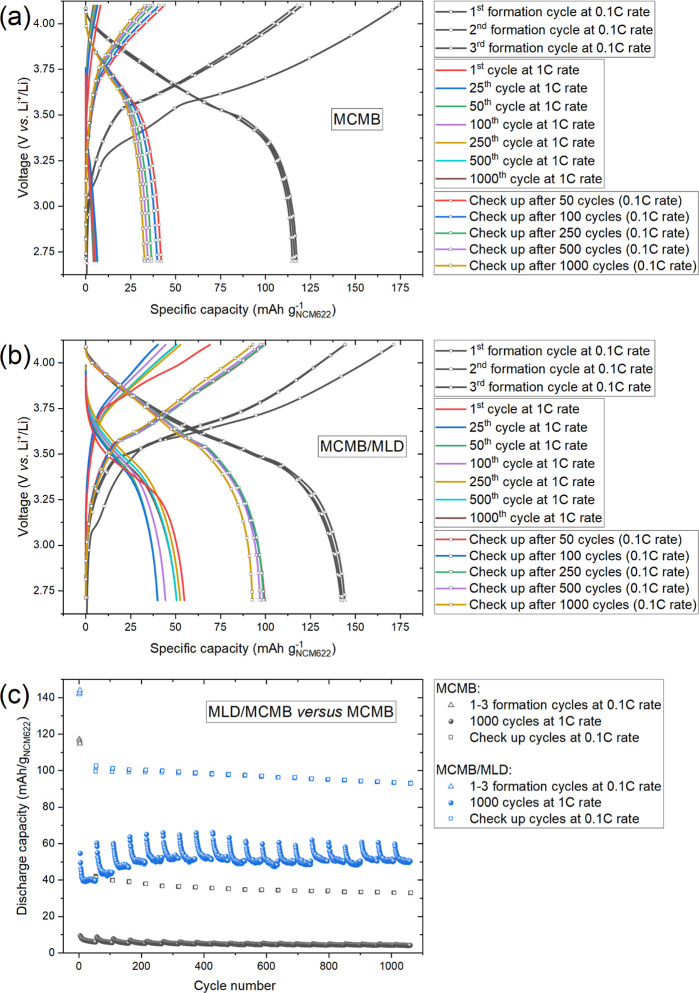
Galvanostatic charge–discharge profiles (a,b) and the discharge capacity versus cycle number dependences (c) recorded for the full cells assembled from NCM_622_ commercial cathodes and different anodes: MCMB and MCMB/MLD. After completing 3 formation cycles (0.1 C rate), two different C-rates were applied in a systematic manner: 50 cycles at a 1 C rate followed by 3 checkup cycles at a 0.1 C rate. This protocol was repeated 20 times, giving a total of 1063 cycles.

### 3.4 On the Chemical Transformations Occurring during Art-SEI Formation via the Thermal MLD Method: A Quantitative Assessment of Possible Reaction Pathways

In this section, the most possible reaction routes within the proposed complex MLD process are considered and quantitatively assessed by a computational analysis of the changes in internal energy (Δ*E*). First, it is worth noting both the similarities and differences in the key processes leading to the formation of the Art-SEI using the MLD technology, compared to the mechanism of SEI formation during the electrolytic reduction of the Li-ion-based electrolyte. In both cases, apparently, ring opening and partial polymerization of the EC molecules occur with the formation of intermediate organic and inorganic compounds, which eventually form the SEI. However, in the case of the “traditional” electrochemical reduction, the solvated electrons released at the anode surface act as a reducing agent, and these processes are triggered by a one-electron reduction of organic compounds with the formation of anion radicals, causing free-radical reactions leading eventually to the formation of SEI components.^66^ In contrast, in the processes related to the formation of Art-SEI via MLD, ring opening in EC followed by partial polymerization apparently occurs under the influence of TMA and/or LiO^*t*^Bu through two-electron processes that do not involve the formation of free radicals. Such an important difference must obviously lead to other types of mechanisms: specific reaction pathways occurring in the presence of different MLD-precursors, namely, the strong electrophile TMA and the nucleophile LiO^*t*^Bu, will be further considered separately.

Next, we will be discussing the possible reactions and pathways related to alkyl carbonate solvents (EC, DMC) and their interactions with the MLD-precursors, as well as the role of LiPF_6_ in the process. TMA is a strong Lewis acid and at the same time a very powerful reducing agent. As such, TMA performs reduction via the migration of a pair of electrons along with the methyl group to the electrophilic center of the molecule being reduced, with possible subsequent rearrangements of the resulting products. If the molecules being reduced contain nucleophilic centers, for example, the oxygen atom of the carbonyl group in carboxylic acid esters, then the transfer of the methyl group is preceded by the formation of a complex with a coordination bond between the aluminum atom in TMA and the carbonyl oxygen atom. It has been shown that this is a key process in the caprolactone ring-opening MLD of organic-aluminum oxide polymer films.^67^ While first evaluating and studying EC interactions, we hypothesized that similar processes also occur during the interaction of TMA with the EC, as shown in Scheme 2.

**Scheme 2:**
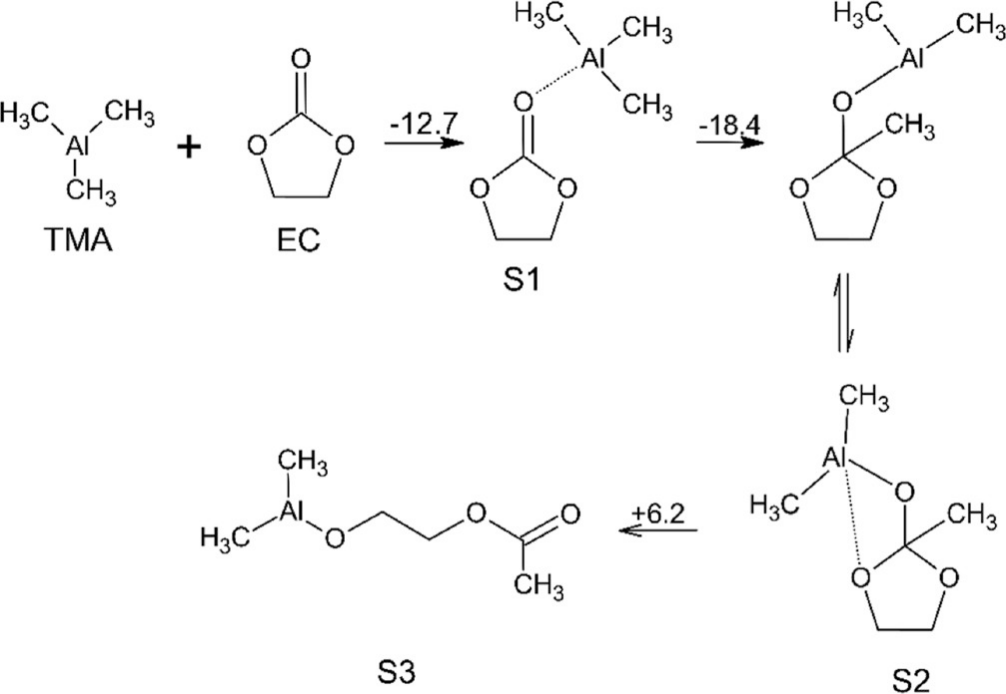
Suggested Reaction Mechanism of TMA with EC^a^

a The numbers above the arrows represent the changes in energy in kcal/mol.

The first step in the chemical process is the coordination of the Al atom from TMA to the carbonyl oxygen of EC. According to our calculations, this is an energetically favorable reaction, with Δ*E* = −12.8 kcal/mol. It also follows from our calculations that the subsequent process of transferring the methyl group from the Al atom in the TMA to the electrophilic carbon atom in the carbonyl group is also energetically very favorable with an Δ*E* of −18.4 kcal/mol. An examination of the geometric structure of the product formed in this pathway showed that the aluminum atom in it forms an additional coordination bond with one of the oxygen atoms in the ring, which forms the prerequisites for subsequent rearrangement with a four-ring redistribution of bonds with a break in the ring and the formation of a noncyclic intermediate containing the −Al­(CH_3_)_3_ group on one side and the −O­(C=O)­CH_3_ ester group on the other. A similar process has been described for ring-opening polymerization of l-lactides catalyzed by aluminum alkyl catalysts.^68^

It is obvious that when these groups belong to different molecules, an exothermic reaction can occur between them, leading to polymerization with the formation of “alucones”. According to our calculations, the ring-opening rearrangement is weakly endothermic with Δ*E* = +6.2 kcal/mol, and therefore, its feasibility may be due to the elevated reactor temperature (225 °C) and the strong exothermic nature of the subsequent polymerization reaction.

Furthermore, the literature describes the polymerization of EC under the simultaneous action of a Lewis acid (such as Li-salt) and a base (such as R–O^–^).^69^ In this case, the Lewis acid is coordinated by the carbonyl oxygen atom, whereas the Lewis base can attack two electrophilic centers (the carbonyl oxygen and the carbon of the CH_2_ group), leading to different ring-opening products and, therefore, different structures of the monomer units of the resulting copolymer. The reaction of the first type is endothermic, and therefore, its feasibility depends on the concurrent reaction of the second type, accompanied by the release of the entropically very favorable CO_2_ molecule. We hypothesized in Scheme 3 the proposed mechanism, in which a similar process might occur when LiO^*t*^Bu interacts with EC: Li^+^ being the Lewis acid and ^*t*^Bu–O^–^ being the Lewis base.

**Scheme 3:**
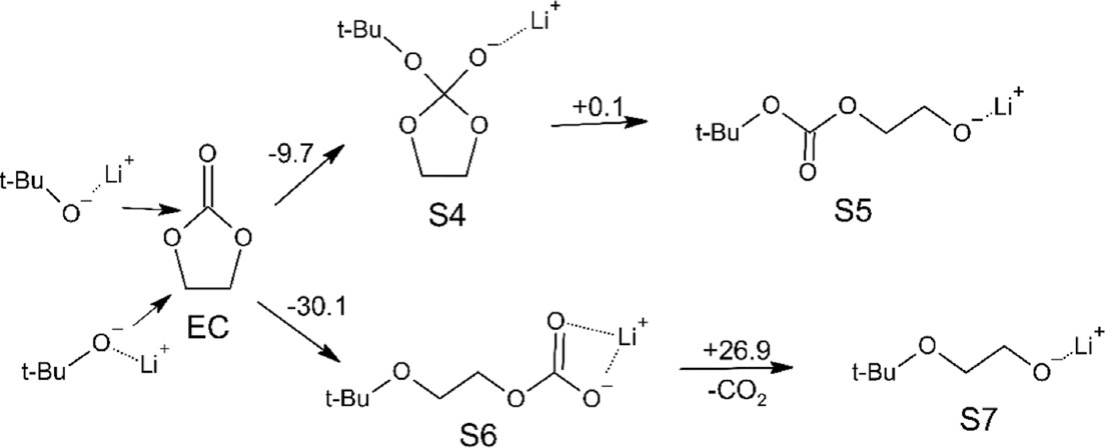
Hypnotized Reaction Pathways of LiO^*t*^Bu Reaction with EC^a^

a The numbers above the arrows represent the change in energy in kcal/mol.

The calculations showed that chemical transformations caused by LiO^*t*^Bu attacks in two pathways are energetically favorable. In the first one, as a result of the attack of the ^*t*^BuO^–^ group on the carbonyl carbon, a ring adduct is formed (**S4**) with Δ*E* = −9.7 kcal/mol; and, in the second pathway (attack on the CH_2_ group), a ring opening occurs (**S6**) with Δ*E* = −30.1 kcal/mol. Thus, the second pathway with subsequent ring opening is energetically significantly more favorable compared to attacking the carbonyl group with the formation of a ring adduct. It also follows from our calculations that adduct **S4** can be in equilibrium (Δ*E* = +0.1 kcal/mol) with the open-chain form (**S5**), which can potentially react with a fresh new EC molecule, according to a similar scheme (Scheme S1 in Supporting Information), where the EtOLi compound is used instead of **S5** and **S7** compounds (as shown in Scheme 3) to simplify the calculations. The resulting products **S9** and **S11** (in Scheme S1) can also react with a new portion of EC molecules, thus leading to a polymerization process. Although this process may lead to polymer formation, its feasibility seems unlikely due to a significant energetic preference for the alternative process, leading to the formation of a stable lithium salt (**S6** and **S10**). Since similar lithium alkyl carbonates R–OC­(=O)­O–Li are part of the organic layer of a natural SEI, it is logical to assume that they could also be a part of the Art-SEI. Our calculations indicate that further decomposition processes of **S6** and **S10**, with a release of CO_2_ and the formation of **S7** and **S11** intermediates, leading to the growth of the polymer chain, are energetically unfavorable with Δ*E* = +26.9 kcal/mol.

Now, one may consider the role of LiPF_6_ (Li-salt presented in the LP-30 electrolyte) in the MLD process: the release of CO_2_ and the formation of **S7** and **S11** intermediates cannot be completely excluded due to the possibility of a reaction of the resulting products with the thermal decomposition products of LiPF_6_ to form products that are typically included in the SEI. It can be assumed that the energetically favorable reaction between PF_5_, which is formed during a thermal decomposition of the LiPF_6_, with the R–O^–^ and R–C­(=O)­O^–^ bases, is an additional factor leading to the termination of the polymerization pathways and the formation of Li-, P-, and F-containing compounds in the Art-SEI.

Now, let us discuss the interactions of DMC (the second solvent in the electrolyte) with the TMA. Scheme S2 (Supporting Information) is quite similar to Scheme 2, but now it replaces EC with DMC: it is easy to see that the energy characteristics of the given processes are close to those shown in Scheme 2. The main difference is that in the case of DMC, there is no ring opening, and no products that may lead to polymerization are being formed. Putative reactions of DMC with lithium *tert*-butoxide (LiO^*t*^Bu) and EtOLi (which represents RCH_2_OLi with different R) are shown in Schemes S3 and S4 (Supporting Information), respectively. The first thing that is obvious when considering these schemes is that in both cases, the most energetically favorable processes lead to the formation of **S20**—lithium methyl carbonate (LMC). Note that LMC is also an important component of conventional “natural” SEI, which is formed on the anode surface in lithium-ion cells also from DMC reduction, but by a completely different mechanism through electrochemical reduction. Comparing the two schemes, it can also be noted that the chemical transformation involving the attack of LiO^*t*^Bu on the carbonyl carbon atom to form adduct **S16** is significantly less energetically favorable compared to the similar process involving EtOLi, apparently due to the steric hindrance caused by the presence of three methyl groups in the *tert*-butyl group. When attacking the electrophilic methyl group, leading to the formation of the above-mentioned LMC, the reaction with LiO^*t*^Bu is also less energetically favorable compared to EtOLi, but the difference in this case is already small due to smaller steric hindrances. All of this is in good agreement with the well-known concept from synthetic organic chemistry on the weaker nucleophilicity of ^*t*^BuO^–^ compared to that of RCH_2_O^–^. It is logical to assume that some of the intermediates obtained from DMC may be involved in processes related to EC, which open the door to additional new and complex pathways.

### 3.5 Durability and Longevity of the Art-SEI Exposed to an Open-Air Environment

One of the most valuable achievements of this research is the development of an Art-SEI film that is resilient, demonstrating high resistance to oxidation and hydration in the presence of atmospheric oxygen and moisture, as will be demonstrated and discussed shortly. As a starting point, we relied on the results described in Section 3.3, namely, that the coating manifested robustness and did not lose its electrochemical properties when using the water-based binder processing for composite electrode fabrication and production. This means that the lithium in the coating film is in a bound state, providing sufficient protection against either dissolution or irreversible oxidation in the presence of the aqueous media. We attributed this protection to the use of an Al-based cross-linker, namely, TMA molecules. A similar protective effect in LIBs, where the presence of aluminum-containing species in an ultrathin film increases the corrosion resistance many times over, has been previously reported in the literature.^70^ Interestingly, a uniform distribution of aluminum in the cross section of the MLD layer was demonstrated (Figures 4h and S6).

Continuing with this logic, we investigated the capability of Art-SEI designed at the surface of a powdered active material to preserve its electrochemical properties while being long-exposed to an open environment (air and humidity). Undoubtedly, such extraordinary capability, if achieved and reliably proven, would become a breakthrough in the state-of-the-art of Li-ion technologies, opening pathways to considerably improved safety, eliminating transportation and storage challenges, and commercializing various “pre-SEI’ed” active electrode materials.

Specifically, once the Art-SEI layer was applied to the MCMB graphite powder, it was exposed to an atmosphere at controlled conditions of 25 °C temperature and 60% humidity in a well-controlled climate chamber for 2 weeks. Then, composite anode electrodes were fabricated from such air-exposed powder, and the electrochemical measurements were performed in both half-cell and full-cell configurations. These results were analyzed and compared with those obtained for the Li-ion cells produced from the pristine (noncoated) MCMB graphite material, to which the same protocol of air exposure was applied. Notably, the ability of the coating to significantly mitigate irreversible capacity in the initial charge–discharge cycles was preserved in the air-exposed material, which allowed us to substantially minimize Li-ion consumption in the half-cell analysis by more than 82 mAh/g in the first formation cycle (Figure S10).

The encouraging results presented in Figure S10 motivated the move into full-cell long-term cycling analysis (500 cycles), which revealed and confirmed the robustness and preservation of the electrochemical properties of the Art-SEI layer, being pre-exposed to atmospheric oxygen and moisture for 2 weeks (Figure 10).

**Figure 10:**
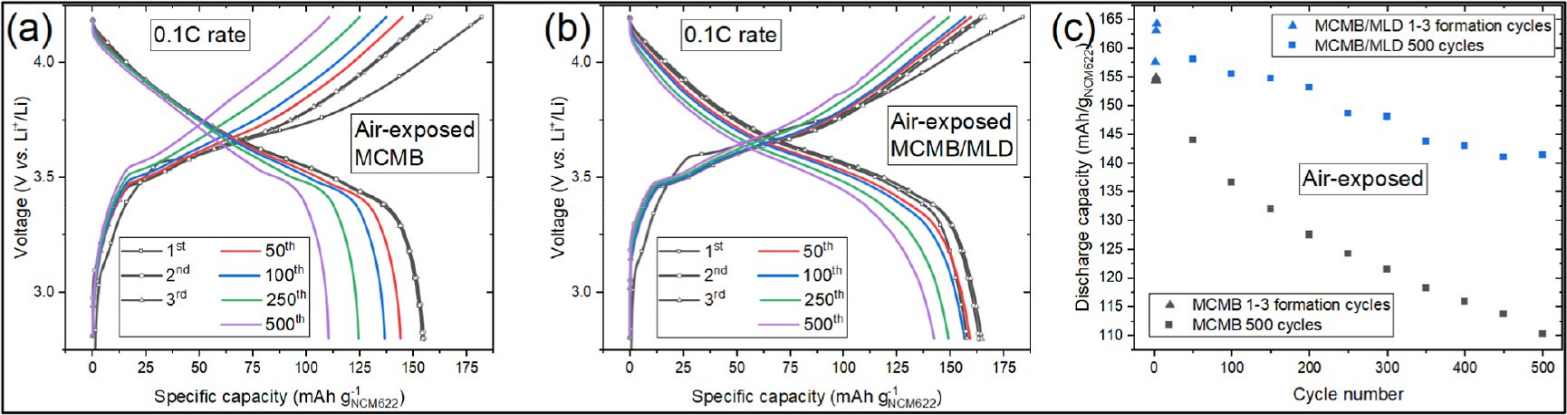
Galvanostatic charge–discharge profiles (a,b) and the corresponding discharge capacity versus cycle number dependences (c) recorded at a 0.1 C rate for the full cells assembled from NCM_622_ commercial cathodes and different anodes: MCMB and MCMB/MLD, both air-exposed for 2 weeks—see explanation in the text.

Both the charge and discharge capacities of the full cells containing the modified and coated Art-SEI MCMB anodes (Figure 10b) were significantly higher than those of the cells utilizing pristine MCMB (Figure 10a) exposed to the same environmental conditions, demonstrating superior and enhanced stability upon cycling. Specifically, throughout the test, the full cells with MCMB/MLD electrodes showed a gradually increasing gain, reaching 31.1 mAh/g by the 500th cycle (Figure 10c). Thus, we can conclude that the application of Art-SEI on graphite powder, as a preconditioning step presented in this study, is a viable option for battery materials manufacturers and it can be applied safely, with no concern for an interaction of the produced Art-SEI film with the environment.

## 4 Conclusions

In this work, a novel air- and moisture-stable Art-SEI thin film was designed and engineered “in vitro”, possessing protective characteristics, with significantly superior electrochemical properties. The film was conformally fabricated onto various electrode substances, such as the MW-CNT tissue, and on the surface of individual particles of powdered MCMB graphite, using the MLD-coating method performed in fixed and fluidized bed reactors, respectively.

Specifically, in this new MLD process, a commercial LIBs’ electrolyte (LP-30), a cross-linker (TMA), and a Li-ion source (LiO^*t*^Bu) were used as precursors. A systematic comparison of the designed Art-SEI to the electrolytically formed natural SEI was carried out, characterizing the film composition and performance of Li-ion cells, namely, cycle life, interfacial kinetics, C-rate performance, initial capacity loss, and Coulombic efficiency. The most possible reaction pathways of this unique MLD process, enabling the formation of the robust Art-SEI, were discussed and quantitatively assessed by computational analysis of the changes in internal energy (Δ*E*), enabling the identification of the most energetically favorable constituents of the Art-SEI. The obtained Art-SEI layer demonstrated enhanced cycling performance in half-cells and full cells, essentially mitigating the irreversible capacity loss in the batteries in the early stages of the formation cycles. These characteristics are mainly attributed to the formation of Li-carbonate, as well as Al and Li alkyl carbonates at the carbon surfaces.

Among the relevant pending issues to be considered are the identification and comparative study of additional and possibly effective organometallic cross-linkers. Moreover, the application of sodium *tert*-butoxide instead of lithium *tert*-butoxide would extend the present study to Na-ion batteries as well. This work also provides an opportunity to implement such an MLD method, utilizing the electrolyte with a cross-linker and Li-ion source as precursors, enabling molecular engineering and designing of a robust cathode electrolyte interphase (CEI), redeeming more capacity in the form of maximizing the available Li-ions for the reversible electrochemical delithiation–lithiation processes. Thus, one can in principle implement Art-SEI and Art-CEI film coatings on both the anode- and cathode-active material powders, respectively, achieving eventually higher cell energy and enhanced stability upon cycling.

## Supplementary Material



## Notes

Additional experimental details, materials, data, and methods, including schemes of mechanisms (PDF)
