# Supplementary material for: Artificial Solid Electrolyte Interphase Developed In Vitro by Tailoring Molecular Layer Deposition of a Li-Ion-Containing Electrolyte on Carbonaceous Anode Materials
Source: ACS Appl Mater Interfaces. 2025 Sep 16;17(39):54668–87. doi: 10.1021/acsami.5c08880 (PMC12492323; doi:10.1021/acsami.5c08880)
Supplement: Supplementary file 1 [file am5c08880_si_001.pdf]

Supporting Information

Artificial Solid Electrolyte Interphase Developed *in-vitro* by Tailoring Molecular Layer Deposition of Li-ion Containing Electrolyte on Carbonaceous Anode Materials

Roman G. Fedorov[1], Jonas Schlaier[2], Nickolay Solomatin[1], Mahmud Auinat[1], Igor Baskin[1], Christian Heubner[2], Alexander Michaelis[2,3], Yair Ein-Eli [1,4,5]**

[1] Department of Materials Science and Engineering
Technion-Israel Institute of Technology
Haifa 320003, Israel
Corresponding author's E-mail: einelі@technion.ac.il

[2] Fraunhofer Institute for Ceramic Technologies and Systems IKTS
01277 Dresden, Germany
Corresponding author's E-mail: alexander.michaelis@ikts.fraunhofer.de

[3] Institute of Materials Science
TU Dresden
01062 Dresden, Germany

[4] Grand Technion Energy Program
Technion-Israel Institute of Technology
Haifa 320003, Israel

[5] Israel National Institute for Energy Storage (INIES),
Technion-Israel Institute of Technology
Haifa 320003, Israel

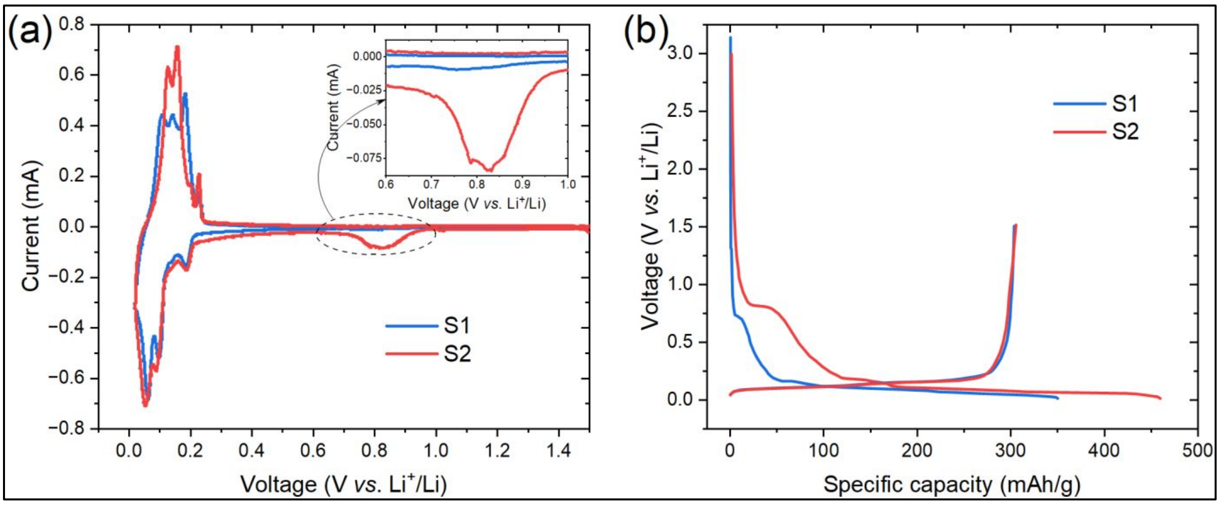

Figure S.1. The sequential steps manifested while utilizing 2 different graphite composite anodes [named as "S1" (blue curves) and "S2" (red curves)] being initially negatively polarized (towards intercalation steps; discharged), followed by a positive potential step (towards deintercalation; charged) imposed in half-cells (vs. Li metal) in nonaqueous electrolytes (all potentials are being quoted vs. Li/Li^+ RedOx couple): (a) at an extremely slow scan rate (5 µV/s) cyclic voltammetry, where the potential is being scanned from open circuit potential (OCP of 3-3.3 V) down to 20 mV and back to 1.5 V. The CV in the Figure shows only the potential window of interest (1.5 to zero V); (b) a constant current in slow rates operational mode, completing a discharge process (intercalation step), in a current corresponding to an expected full lithiation in a time frame of 10 hours (0.1 C) down to a potential 20 mV and back to 1.5V.

SEI's capacity losses would depend on the following parameters: the type of carbon anode and its surface area; electrolyte composition; binder type; rate of charging and current collector type. * The SEI's capacity consumption (and therefore the loss of Li-ion inventory at the cathode) may vary from five to even 400% of the 372 mAh/g carbon's theoretical capacity.

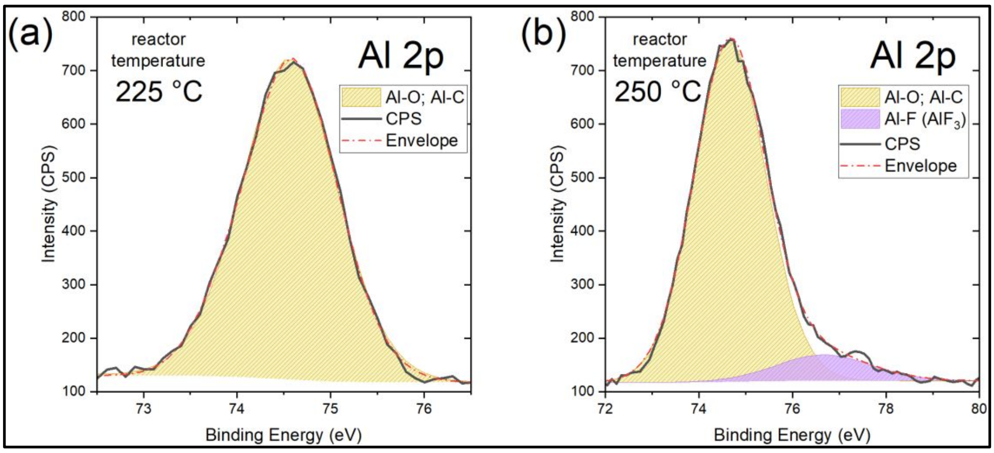

Figure S.2 XPS spectra of Al 2p region of the MCMB/MLD graphite powder surface obtained at different MLD-reactor temperatures of 225 °C (**a**) and 250 °C (**b**).

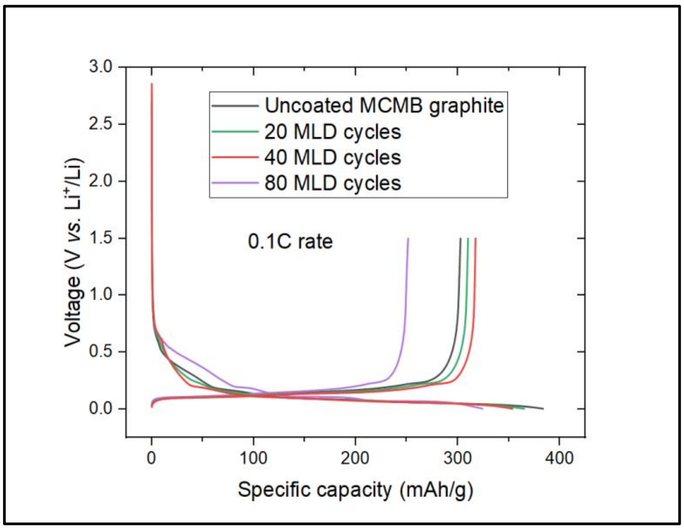

Figure S.3. Galvanostatic charge/discharge profiles of the first cycle at 0.1C rate, recorded in a half-cell configuration for the MCMB and different MCMB/MLD anodes: 20, 40 and 80 MLD cycles.

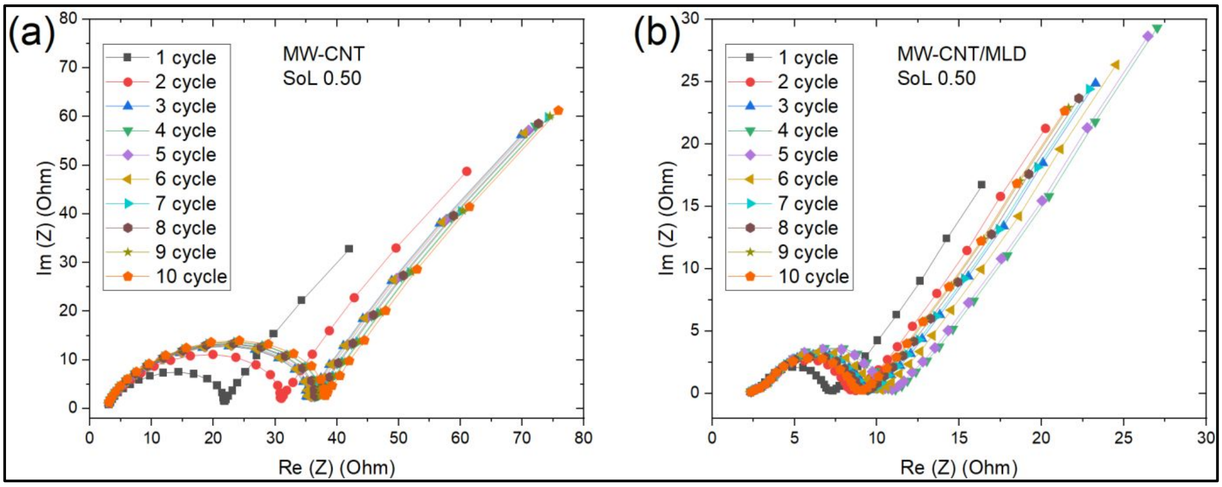

Figure S.4. Electrochemical impedance spectroscopy (EIS) profiles of the 10 initial cycles recorded for the pristine (a) and MLD-coated (b) MW-CNTs electrodes at 0.50 state of lithiation (SoL).

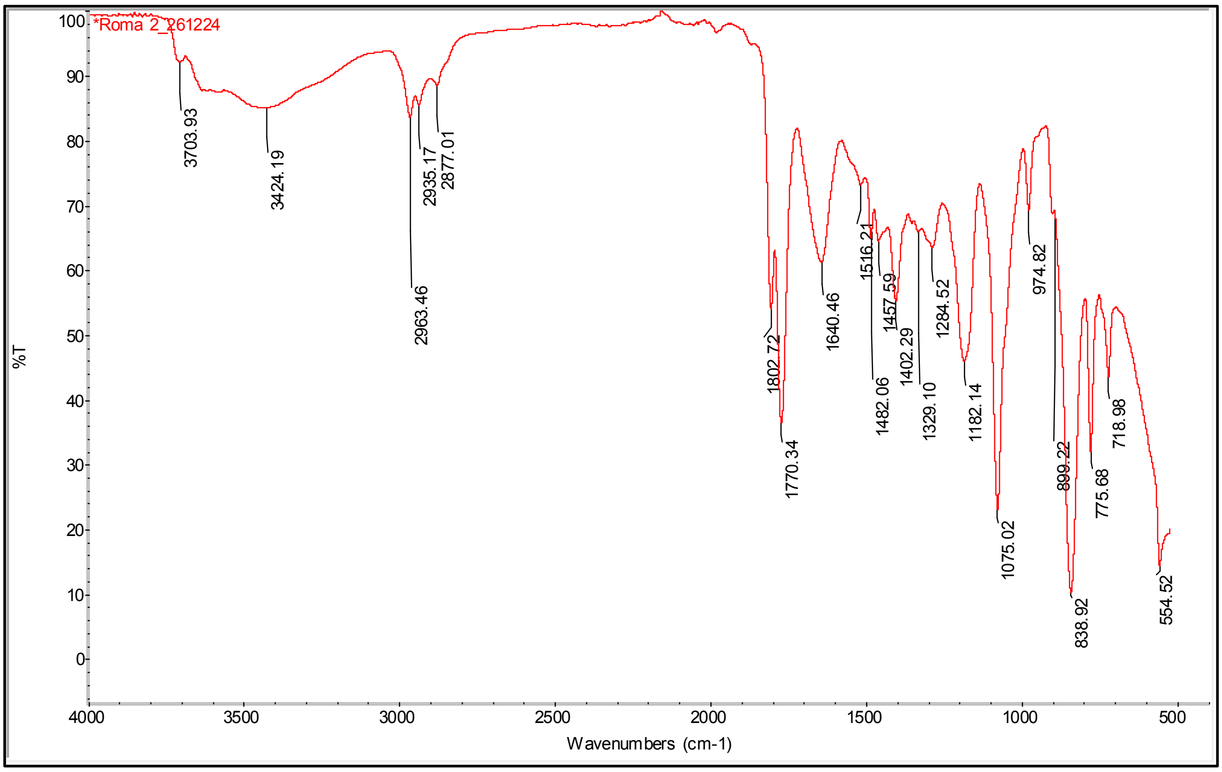

Figure S.5. FTIR spectrum of the MCMB/MLD graphite powder surface obtained on the same array of MCMB/MLD microparticles presented at Figure 4b.

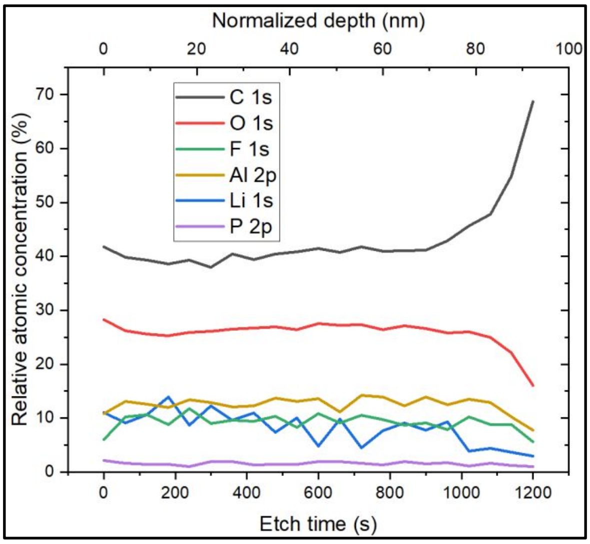

Figure S.6 XPS depth profiling of C 1s, O 1s, Al 2p, Li 1s, P 2p and F 1s regions of the MCMB/MLD graphite powder surface obtained on the same array of MCMB/MLD microparticles presented in Figure 4b.

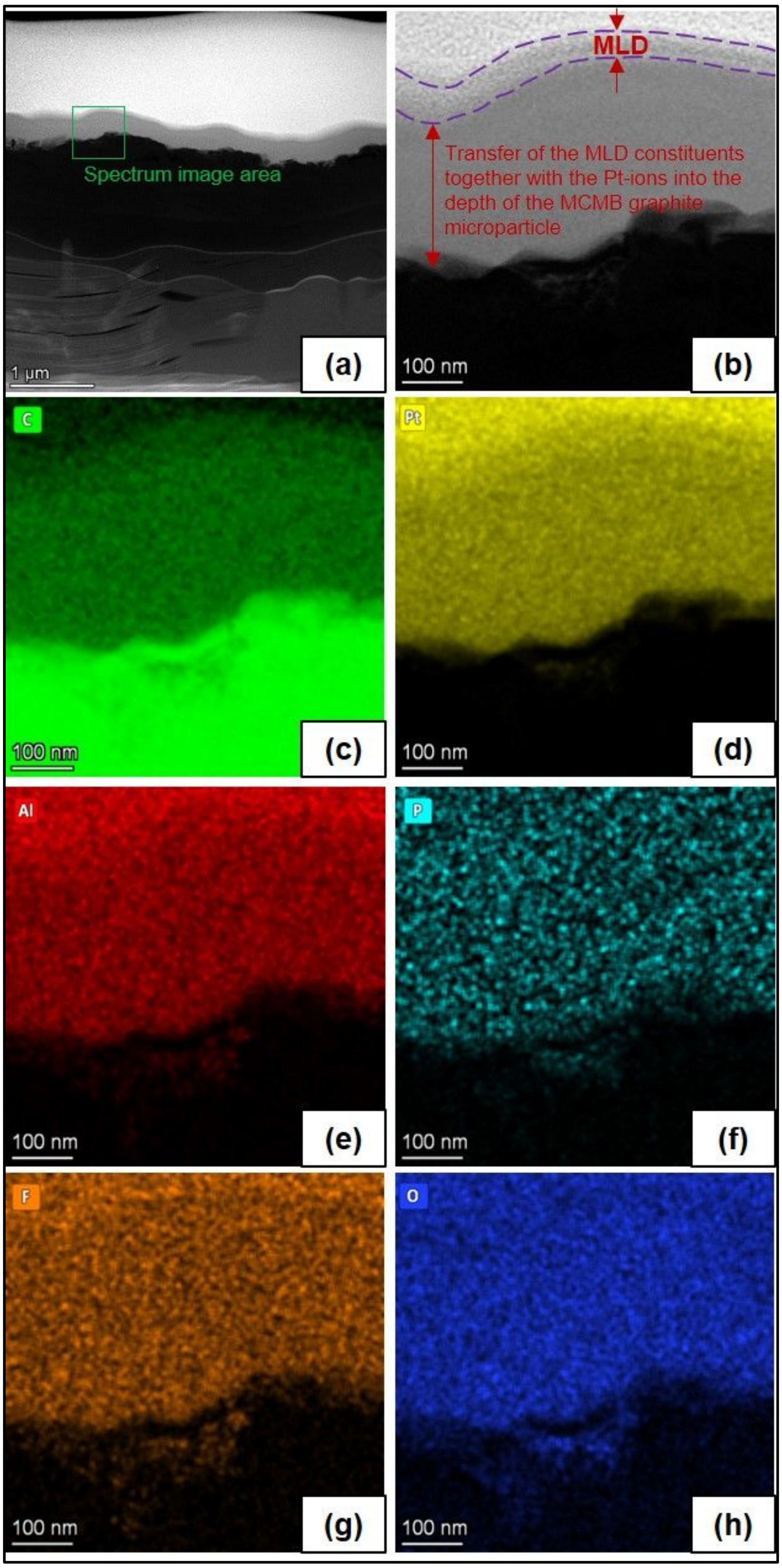

Figure S.7. SEM image showing the localization of cross-sectional TEM structural and elemental analysis of the MLD/MCMB interphase (a): the HAADF-STEM image (b) and corresponding STEM-EDS single-element mapping images (c-h) of the MLD/MCMB interphase cross-section recorded for the different elements: C (c), Pt (d), Al (e), P (f), F (g), and O (h).

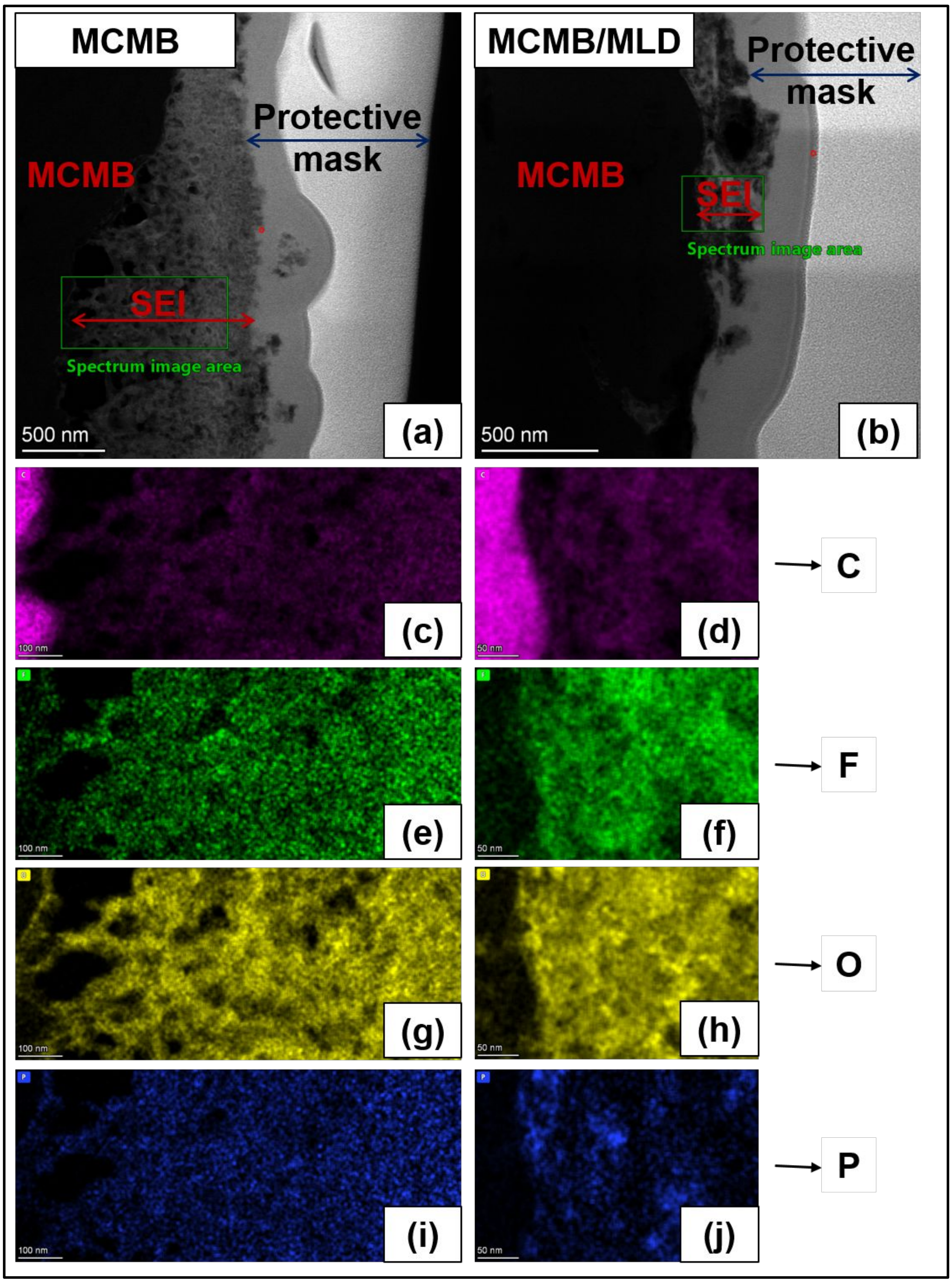

Figure S.8. TEM images of the interface cross-section of graphite species extracted from the MCMB (a) and MCMB/MLD (b) composite electrodes that had been subjected to 100 charge-discharge cycles at 0.1C rate; and corresponding STEM-EDS single-element mapping images (c-j) of the MLD film cross-section recorded for the different elements: C (c-d), F (e-f), O (g-h), and P (i-j). The spectrum image areas are outlined with green rectangles at (a) and (b).

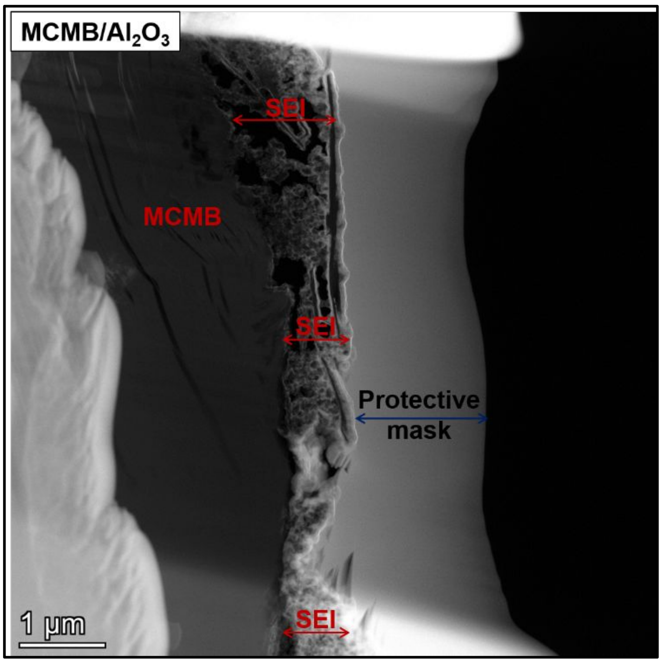

Figure S.9. TEM images of the interface cross-section of graphite specimen extracted from the MCMB/Al_2O_3 composite electrode that had been subjected to 100 charge-discharge cycles at 0.1C rate.

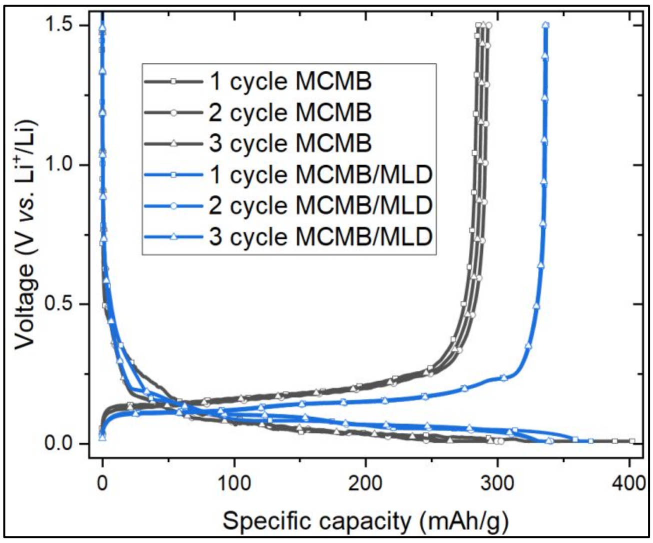

Figure S.10. Galvanostatic charge/discharge profiles of the initial three cycles at 0.1C rate, recorded for the MCMB and MCMB/MLD anodes in a half-cell configuration, both air-exposed (25 ^{o}C, 60% humidity) for 2 weeks.

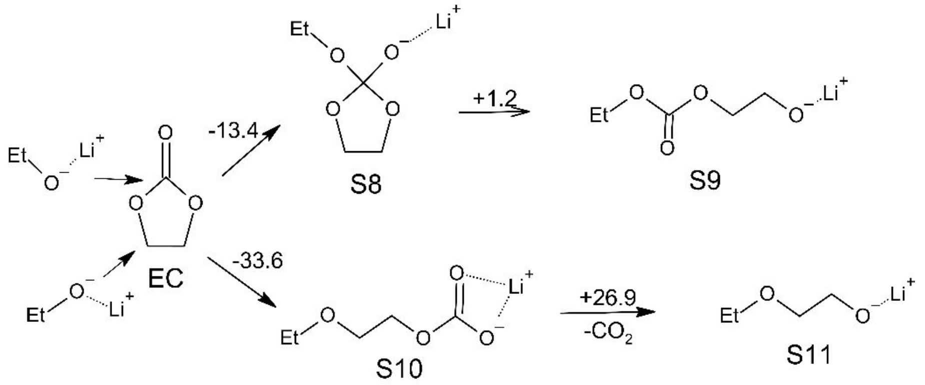

Scheme S.1. Suggested reaction of EtOLi with EC. Here, the EtOLi compound is used instead of **S5** and **S7** compounds (Scheme 3) to simplify the calculations. The numbers above the arrows represent the changes in energy in kcal/mol.

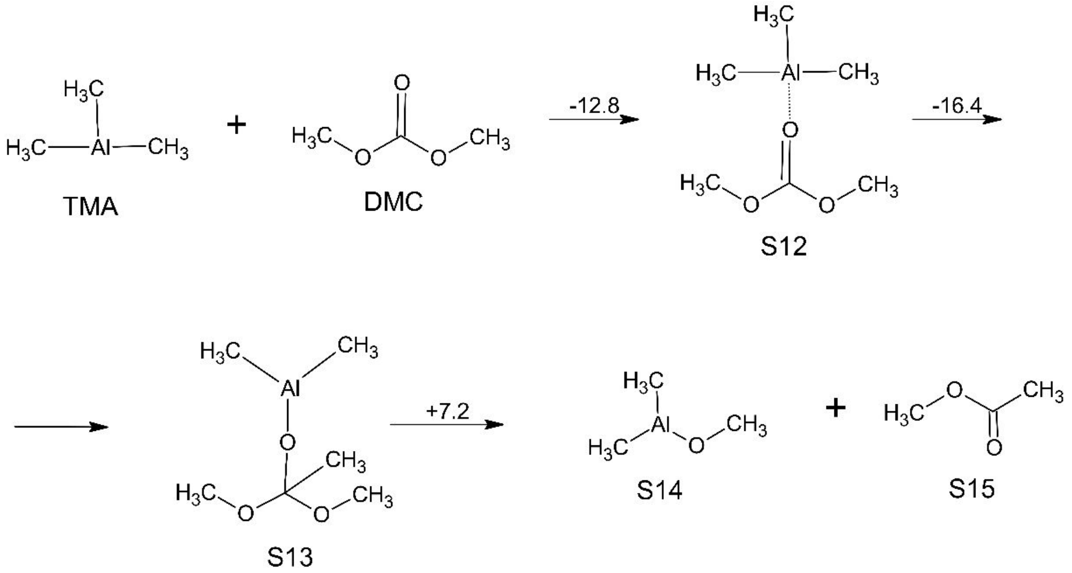

Scheme S.2. Possible reaction pathway of TMA with DMC. The numbers above the arrows represent the changes in energy in kcal/mol.

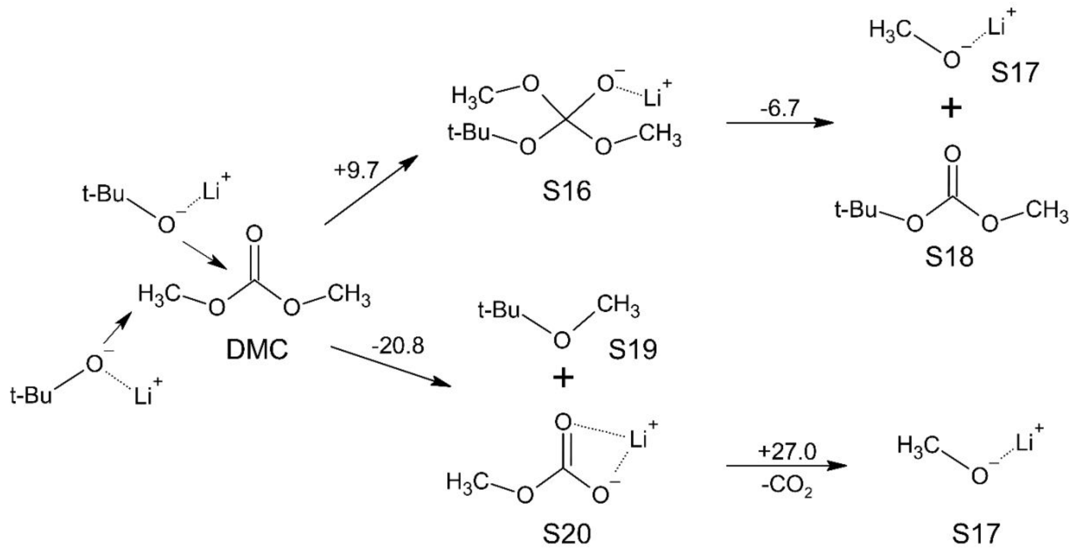

Scheme S.3. Possible reaction of t-BuOLi with DMC. The numbers above the arrows represent the change in energy in kcal/mol.

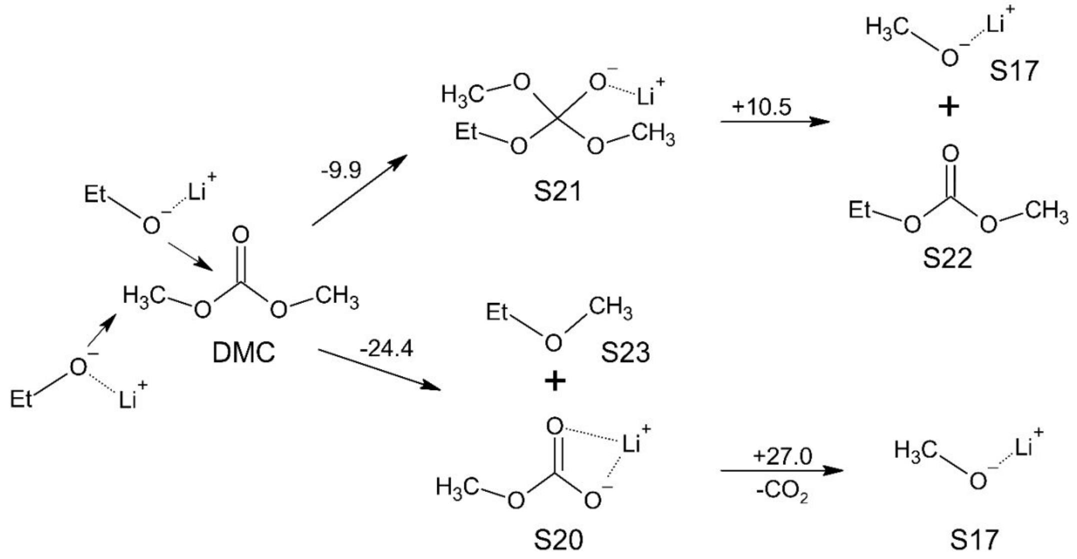

Scheme S.4. Suggested reaction of EtOLi with DMC. The numbers above the arrows represent the change in energy in kcal/mol.
